# Transient Mitochondria Dysfunction Confers Fungal Cross-Resistance against Phagocytic Killing and Fluconazole

**DOI:** 10.1128/mBio.01128-21

**Published:** 2021-06-01

**Authors:** Sofía Siscar-Lewin, Toni Gabaldón, Alexander M. Aldejohann, Oliver Kurzai, Bernhard Hube, Sascha Brunke

**Affiliations:** a Department of Microbial Pathogenicity Mechanisms, Hans Knoell Institute, Jena, Germany; b Barcelona Supercomputing Centre (BSC-CNS), Barcelona, Spain; c Institute for Research in Biomedicine (IRB Barcelona), The Barcelona Institute of Science and Technology, Barcelona, Spain; d Catalan Institution for Research and Advanced Studies (ICREA), Barcelona, Spain; e Institute for Hygiene and Microbiology, Julius-Maximilians-University, Würzburg, Germany; f Institute of Microbiology, Friedrich Schiller University, Jena, Germany; Geisel School of Medicine at Dartmouth

**Keywords:** fungal infection, *petite*, cross-resistance, antivirulence

## Abstract

Loss or inactivation of antivirulence genes is an adaptive strategy in pathogen evolution. Candida glabrata is an important opportunistic pathogen related to baker’s yeast, with the ability to both quickly increase its intrinsic high level of azole resistance and persist within phagocytes. During C. glabrata’s evolution as a pathogen, the mitochondrial DNA polymerase CgMip1 has been under positive selection. We show that *CgMIP1* deletion not only triggers loss of mitochondrial function and a *petite* phenotype, but increases C. glabrata’s azole and endoplasmic reticulum (ER) stress resistance and, importantly, its survival in phagocytes. The same phenotype is induced by fluconazole and by exposure to macrophages, conferring a cross-resistance between antifungals and immune cells, and can be found in clinical isolates despite a slow growth of *petite* strains. This suggests that *petite* constitutes a bet-hedging strategy of C. glabrata and, potentially, a relevant cause of azole resistance. Mitochondrial function may therefore be considered a potential antivirulence factor.

## INTRODUCTION

Human-pathogenic fungi remain an underestimated threat in global health, and the mortality rates of fungal infections worldwide are higher or similar to deaths due to malaria or tuberculosis (1, 2). *Candida* species are among the most important human fungal pathogens and cause millions of mucosal and life-threatening systemic infections each year (1). Candida glabrata has become the second most common *Candida* species for immunocompromised patients, surpassed only by C. albicans as the primary cause of candidiasis (3). However, most of the well-characterized pathogenicity mechanisms of C. albicans are not shared by C. glabrata, and unlike the former, C. glabrata does not cause significant host cell damage or elicit strong host immune responses (4). Among the main clinically relevant attributes and pathogenic traits of C. glabrata are rather a high intrinsic resistance to azole antifungals and an ability to survive for a long time and replicate within mononuclear phagocytes (4–6). Its redundant antioxidative stress mechanisms, combined with its ability to modify the phagosomal pH, may partially account for the remarkable ability to survive phagocytosis by macrophages (7–9). These facts have led to the speculation that C. glabrata may take advantage of these immune cells to succeed as a pathogen and disseminate within the host (6).

Among the strategies that confer pathogenicity, the loss or inactivation of certain genes, termed antivirulence genes, is common in pathogenic microorganisms (10); cellular pathways and functions that are normally advantageous for the microbe can become superfluous or even disadvantageous under infection conditions, and the loss or inactivation of their encoding genes becomes adaptive during infection. Several examples of such antivirulence factors are known in human-pathogenic fungi, and many more are likely to exist (11).

C. glabrata is more closely related to the brewer’s yeast Saccharomyces cerevisiae than to C. albicans (12) and clusters with members of the *Nakaseomyces* group, a genus that includes other environmental and human-associated species (5). In a systematic genomic comparison within this group, four genes showed hallmarks of positive selection in C. glabrata (5). These genes exhibit a relatively high ratio of nonsynonymous to synonymous mutations (*d_N_/d_S_*), indicating positive selection during the diversification of C. glabrata as a species. Therefore, they might be involved in C. glabrata’s specific adaptation to the human host. The gene with the highest *d_N_/d_S_* ratio (3.40) among them is *CgMIP1*, an orthologue of a mitochondrial DNA (mtDNA) polymerase in S. cerevisiae (5).

mtDNA encodes subunits of the respiratory complexes, which are involved in the production of ATP during oxidative phosphorylation. The consequences of mtDNA loss have been well described in S. cerevisiae and C. glabrata, which, unlike other pathogenic yeasts such as C. albicans or Cryptococcus neoformans (13, 14), are known as *petite*-positive yeasts, since they are able to grow without mtDNA. The *petite* phenotype is characterized by loss of mitochondrial function due to complete (rho^0^) or partial loss (or presence of deleterious mutations) of mtDNA (rho^–^) (15). This phenotype is characterized by the namesake small colonies, slow growth, inability to use nonfermentable carbon sources, activation of the transcription factor Pdr1, and upregulation of its targets *CDR1* and *CDR2*, which code for ABC efflux pump transporters (13). This upregulation confers high resistance to azole antifungals (16–18). Indeed, the *petite* phenotype can be obtained by incubation with high concentrations of azole or ethidium bromide (17, 19, 20). Ethidium bromide is known to inhibit mtDNA synthesis and degrade the existing mtDNA (20), but how azoles trigger mitochondrial dysfunction is not entirely clear. Azole treatment is known to trigger a temporary loss of mitochondrial function (21), and the few clinical *petite* strains of C. glabrata described so far have been mainly isolated from azole-treated patients (19, 22). One of these isolates has been further characterized (23). Surprisingly, these slow-growing isolates showed increased virulence in an animal infection model (23). However, when its parental strain was made *petite* by ethidium bromide treatment, its virulence was instead reduced. The same was found in another study using an ethidium bromide-induced *petite* (24). Thus, the clinical relevance of the *petite* form is still unclear, and its identification from patient samples may even be hindered by its long generation time.

This study investigates the relevance of the presence and absence of mitochondrial function for C. glabrata’s adaptation to the host and its pathogenic potential, as well as the potential role of *CgMIP1* for switching between *petite* and non-*petite* phenotypes. Deletion of *MIP1* results in *petite* forms, but in contrast to the S. cerevisiae *Scmip1*Δ mutant, C. glabrata *Cgmip1*Δ survives phagocytosis by macrophages significantly better than wild-type cells. Importantly, the C. glabrata *petite* phenotype is directly induced in wild-type strains by phagocytosis and leads to increased azole resistance, but also vice versa, with azole-induced *petites* resisting phagocytosis better. This indicates a clinically important positive feedback between two relevant phenotypes, resistance to macrophages and azoles. The clinical relevance of this phenomenon was further corroborated by the detection of a number of *petite* strains in clinical samples.

## RESULTS

### *MIP1* knockout mutants of C. glabrata and S. cerevisiae both show *petite* phenotypes but differ in their survival after phagocytosis.

**FIG 1:**
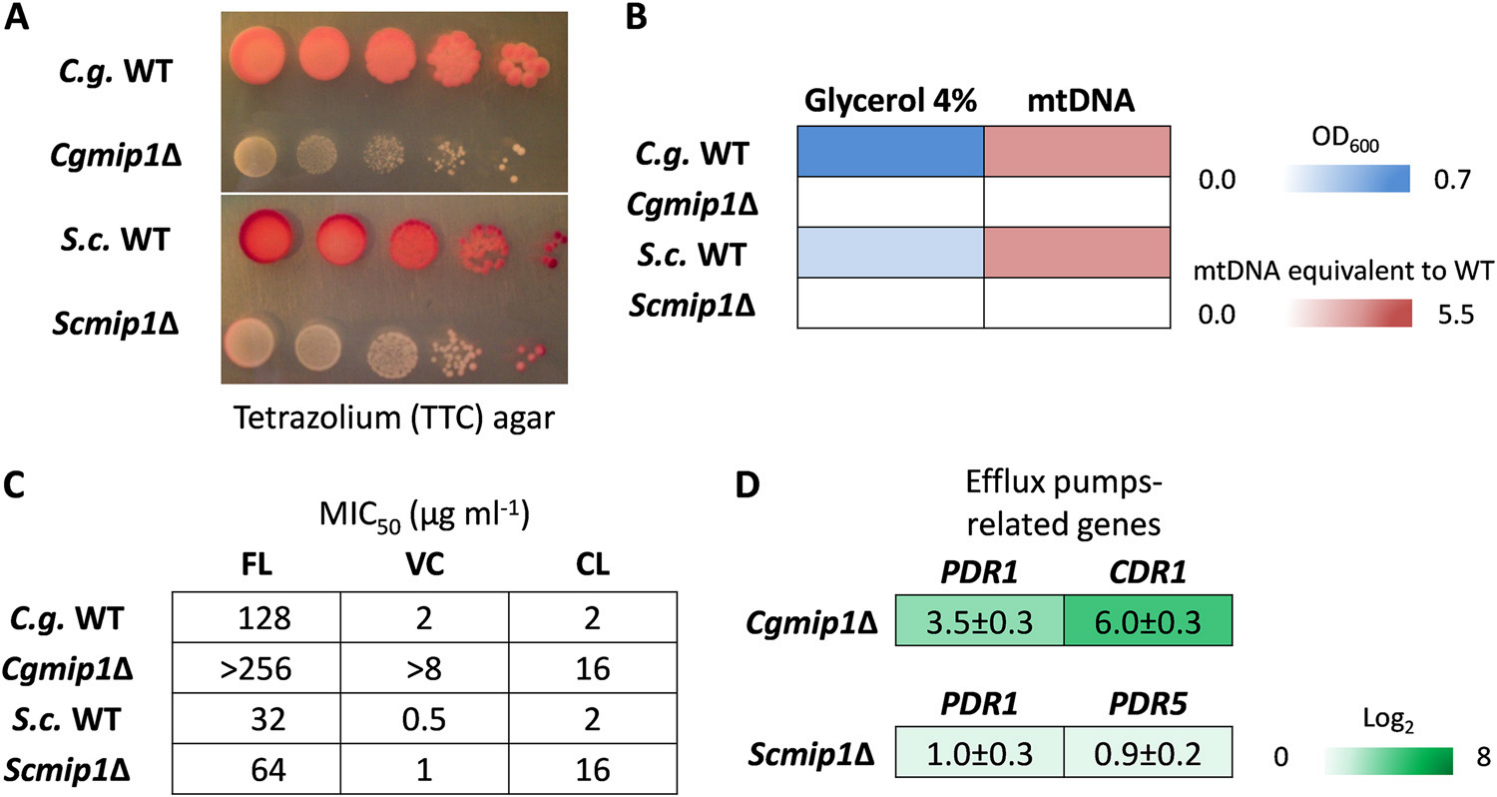
Both *Cgmip1*Δ and *Scmip1*Δ show typical *petite* phenotypes. (A) Small colonies and loss of mitochondrial reductive power, as indicated by lack of tetrazolium dye reduction; (B) lack of growth in alternative carbon sources such as glycerol and absence of mitochondrial DNA (mtDNA) as determined by optical density and qPCR (*n* = 3 for each type of experiment, color by mean); (C) high resistance to azoles, including fluconazole (FL), voriconazole (VC), and clotrimazole (CL), and (D) overexpression of efflux pump-related genes (mean ± SD, *n* = 3 independent experiments with 3 technical replicates each). *C.g.*, C. glabrata; *S.c.*, *S. cerevisiae*.

Since the *MIP1* gene of C. glabrata seems to have been under selective pressure during the pathogen’s evolution, we investigated its functions in virulence-related scenarios. First, we created a deletion mutant of *CgMIP1* (*Cgmip1*Δ) and compared it to a similar deletion of the orthologous gene in S. cerevisiae (*Scmip1*Δ). *ScMIP1* is known to encode a mitochondrial polymerase (*Saccharomyces* Genome Database [SGD], www.yeastgenome.org), and therefore, we first checked whether the mutants show the *petite* phenotype. As expected, both *Cgmip1*Δ and *Scmip1*Δ showed a phenotype typical for *petite* variants (16–18)—small colonies on agar plates and absence of reductive mitochondrial power (Fig. 1A), absence of mtDNA, and lack of growth in nonfermentable carbon sources (Fig. 1B). Moreover, they showed high resistance to azoles (Fig. 1C) and high expression of the efflux pump-related genes *PDR1* and *CDR1* (*PDR1* and *PDR5* in S. cerevisiae) (Fig. 1D), again typical features of *petite* variants. These results therefore show that, like its S. cerevisiae counterpart, *CgMIP1* likely encodes a mitochondrial DNA polymerase, and its deletion triggers loss of mtDNA, loss of mitochondrial function, and a *petite* phenotype in both species.

**FIG 2:**
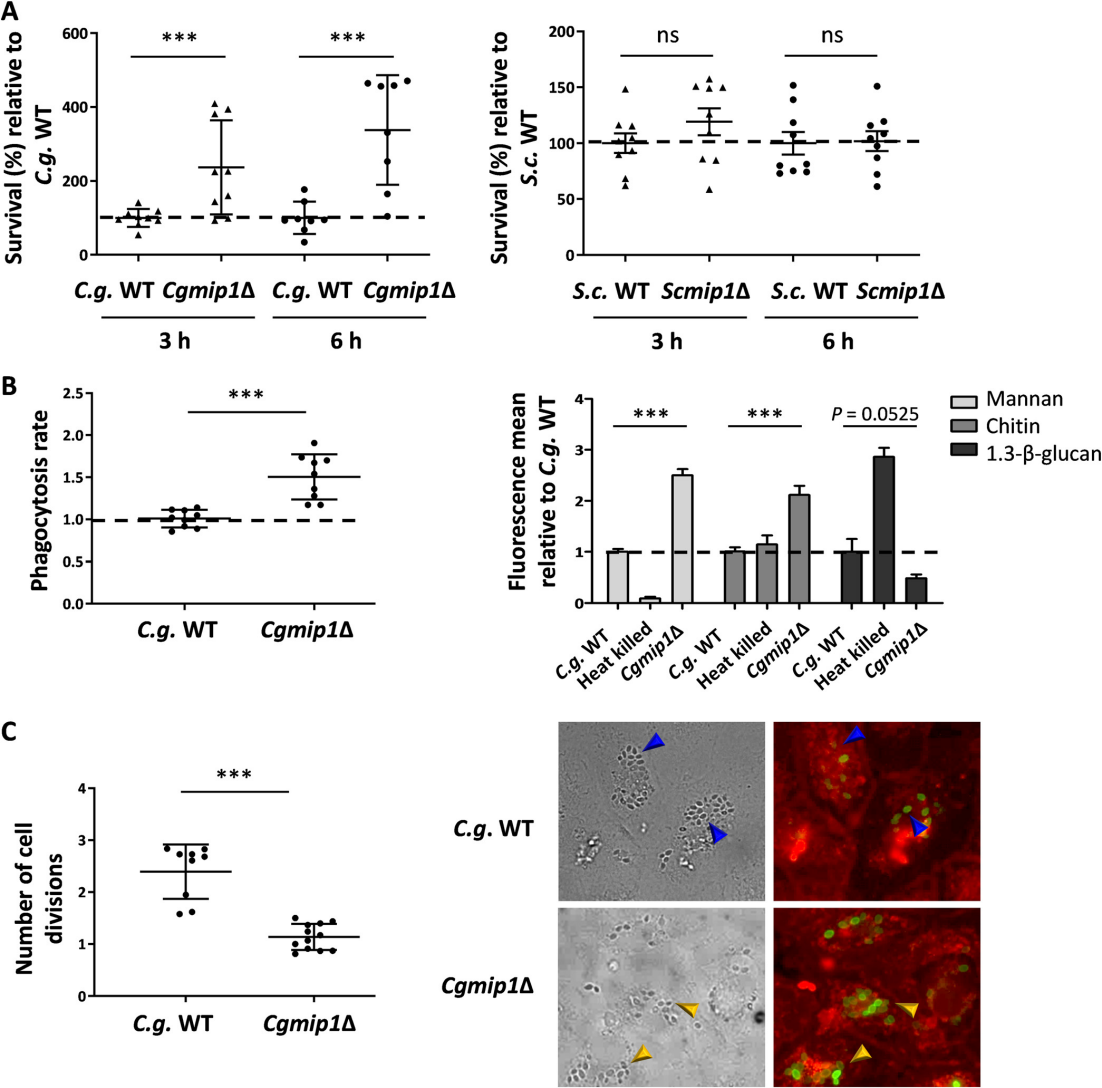
C. glabrata and S. cerevisiae *petite* phenotypes differ in their survival after phagocytosis. (A) *Cgmip1*Δ survives phagocytosis by hMDMs much better than its parental wild type at early time points up to 6 h—in contrast to *Scmip1*Δ, which does not show any change in survival compared to its wild type (mean ± SD, *n* = 9 with 3 different donors in 3 independent experiments; each point represents the mean of 3 technical replicates). (B) *Cgmip1*Δ is taken up at a higher rate than the wild type by macrophages (mean ± SD, *n* = 9 with 3 different donors in 3 independent experiments; each point represents the mean of 3 technical replicates), and its accessible cell wall structures differ from the wild type (mean ± SD, *n* = 3 independent experiments with 3 technical replicates each). (C) In contrast to the wild type, *Cgmip1*Δ does not replicate within the phagosome as shown by FACS (left) and by the lack of FITC-unstained daughter cells (right). These are present in the wild type (blue arrows), in contrast to the mutant, which shows only mother cells (yellow arrows) (representative picture shown). Quantitative data are the mean ± SD; *n* = 12 with 3 different donors in 4 independent experiments; each point represents the mean of 3 technical replicates. Statistically significantly different values (unpaired, two-tailed Student’s *t* test on log-transformed ratios) are indicated by asterisks as follows: ***, *P* ≤ 0.001. *C.g.*, C. glabrata; *S.c.*, *S. cerevisiae*.

To study a possible involvement of *MIP1* in processes relevant for virulence, we subjected *Cgmip1*Δ to phagocytosis by human monocyte-derived macrophages (hMDMs) and analyzed its survival after 3 and 6 h. At those time points, macrophages were lysed, and total CFU were counted after plating on yeast extract-peptone-dextrose (YPD) agar. A significantly higher survival rate of *Cgmip1*Δ was found at both times compared to both the wild-type control and to *Scmip1*Δ (Fig. 2A). In order to confirm that this increased number of surviving intracellular yeasts was indeed due to better survival and not due to differences in phagocytosis rate or internal replication, we measured both parameters. For phagocytosis rates, *Cgmip1*Δ and the wild type were incubated with hMDMs for 1 h, and CFU from supernatant and macrophage lysate were determined. *Cgmip1*Δ cells were taken up at a slightly higher rate compared to the wild type (Fig. 2B), which, however, alone cannot explain the stark increase in the number of intracellular *Cgmip1*Δ especially at 6 h; whereas the *petite* mutant is taken up 1.5 times more than the wild type, the survival of this mutant is up to four times more than that of the wild type after 3 h, and six times more at 6 h.

To gain more insight into the underlying reason for this slightly increased uptake of *Cgmip1*Δ, the exposure of cell wall components was measured by flow cytometry. Significantly higher exposure of mannan and chitin was observed (Fig. 2B), while exposure of β(1→3)-glucan was slightly reduced. Higher surface mannan levels on yeast cells are known to increase the phagocytosis rate (25), and thus, our results show that mitochondrial dysfunction by deletion of *CgMIP1* affects cell wall composition in C. glabrata—in agreement with previous observations (24, 26)—and leads to changes in the phagocytosis rate. In addition, it has been reported that higher surface mannan seems to lead to a decreased killing of C. glabrata (27) in a yet unknown mechanism, from which *Cgmip1*Δ would benefit during phagocytosis. To also directly measure fungal replication within the macrophages, yeasts were fluorescein isothiocyanate (FITC)-stained and incubated with hMDMs for 6 h. This stain is not transferred to daughter cells, and we measured FITC-negative cells in the macrophage lysate by flow cytometry and also visualized them with fluorescence microscopy. According to our FACS data, *Cgmip1*Δ showed a much lower replication rate than its parental strain, and we did not observe any unstained daughter cells by microscopy (Fig. 2C). This is in accordance with the nutrient limitation in the phagosome, where only nonfermentable (and therefore *petite*-inaccessible) carbon sources (carboxylic acids, amino acids, peptides, *N*-acetylglucosamine, and fatty acids) are thought to be available (28–30). This also shows that the wild type is killed much more efficiently than the *petite* strains, resulting in a higher CFU count for the latter despite its lower replication rate within the phagosome.

These results indicate that, although *Cgmip1*Δ *petite* cells are engulfed faster and are largely unable to replicate inside the macrophage, they are killed significantly more slowly in the first hours of immune cell interaction, in clear contrast to nonpathogenic S. cerevisiae.

### The *petite* phenotype emerges from the wild type after phagocytosis.

**FIG 3:**
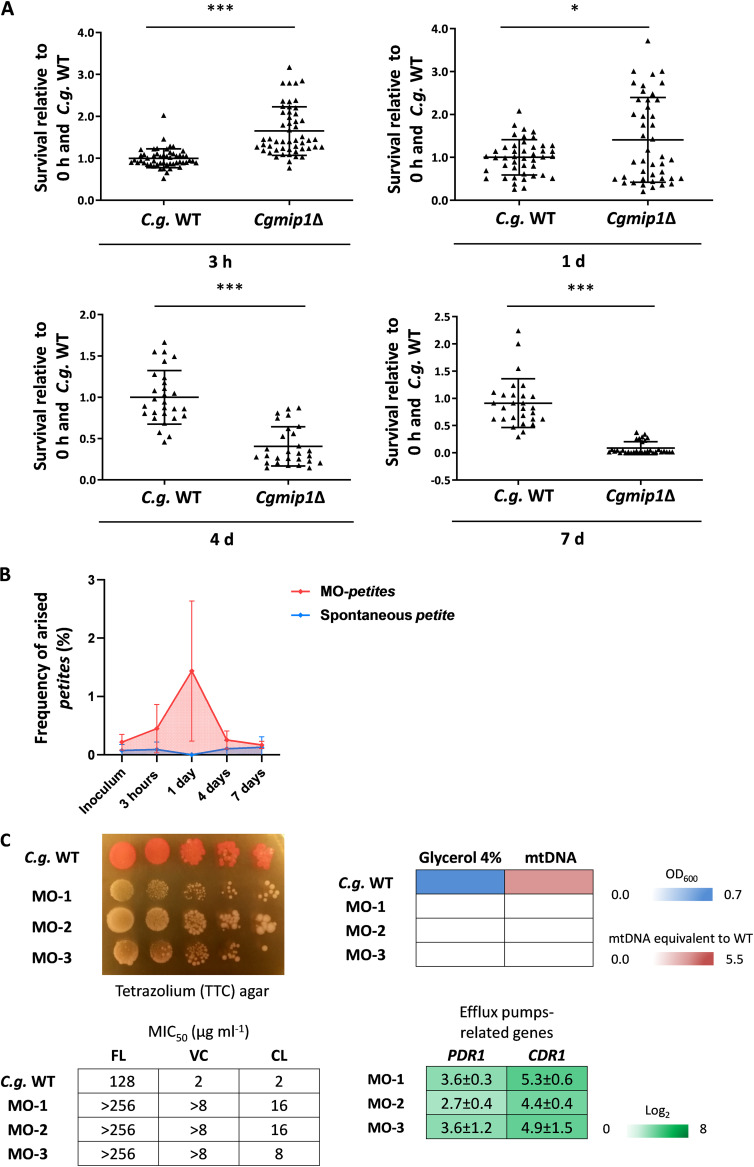
The *petite* phenotype emerges from the wild type after phagocytosis. (A) *Cgmip1*Δ shows a higher rate of survival up to 1 day of coincubation but loses this advantage over extended periods of intracellular existence (*n* = 5 with 1 different donor in each of the 5 independent experiments; each point represents a single survival test). Statistically significantly different values (unpaired, two-sided Student’s *t* test on log-transformed ratios) are indicated by asterisks as follows: *, *P* ≤ 0.05; ***, *P* ≤ 0.001. (B) Cells with the *petite* phenotypes arise from the wild type during coincubation with hMDMs (red) at a much higher rate than the spontaneous appearance of *petite* in RPMI (blue). The time points with the highest frequency of *petite* emergence correspond to the time point of increased fitness of *Cgmip1*Δ during phagocytosis (Red: *n* = 5 with 1 different donor in each of the 5 independent experiments and 4 technical replicates each. Blue: *n* = 3 in 3 different experiments with 6 technical replicates. Mean ± SD of the frequencies of *petite* emergence at each time point). (C) Phenotype characterization reveals the hMDMs-derived strains (MO-1 to -3) to be indeed *petite.* From the top left, small colony forming and lack of mitochondrial reductive power, lack of growth in alternative carbon sources, absence of mitochondrial DNA (mtDNA), high resistance to azoles (all *n* = 3 with mean values or representative picture shown) and overexpression of efflux pump-related genes (mean ± SD, *n* = 3 independent experiments with 3 technical replicates each). FL, fluconazole; VC, voriconazole; CL, clotrimazole; *S.c.*, *S. cerevisiae*.

Our data so far indicate a selective advantage of the *petite CgMIP1* deletion strain during initial interactions with macrophages, despite its inability to replicate within these cells. We therefore analyzed survival of *Cgmip1*Δ and the wild type during long-term residence within macrophages. For this experiment, yeasts were first incubated with macrophages for 3 h, the supernatant was removed, and the yeast-containing macrophages were then incubated for 7 days. Fungal survival was assessed by CFU counting from plated lysate at 3 h, 1 day, 4 days, and 7 days. *Cgmip1*Δ again showed higher survival up to 1 day of coincubation, in full support of our previous results (Fig. 3A). However, at later time points, *Cgmip1*Δ showed a significant decrease in survival. This may be explained by its inability to replicate within the phagosome, leading to a long-term disadvantage. Unexpectedly, we spotted many small colonies during incubation of the wild type. These colonies were more abundant across all observed time points compared to the inoculum, and this was especially true after 3 h and 1 day of incubation (the time points with the best survival of the *petite* strain), with an average frequency of 1.5 × 10^−2^ after 1 day (Fig. 3B). These colonies showed stable small colony formation and lack of growth in glycerol, typical features of the *petite* phenotype. Importantly, this frequency was higher than the spontaneous emergence of *petite* without macrophages at the same time points (Fig. 3B). In addition, some of the colonies which did not grow in glycerol gave rise to a respiratory-competent phenotype, i.e., returned to their original non-*petite* state, when they were plated again on complex medium, with an observed frequency of 5.6 × 10^−2^.

We analyzed several of the stable wild-type-derived *petite* colonies and found—in the majority, but not all of them—a lack of detectable mtDNA. We selected three of these stable colonies from different experiments for further characterization of their *petite* phenotype (Fig. 3C). Besides lacking mtDNA and therefore functional mitochondria, these strains also showed high azole resistance with constitutive expression of efflux pump-related genes, although they were not exposed to azoles, similar to *Cgmip1*Δ (Fig. 3).

We hypothesized that phagosomal reactive oxygen species (ROS) production may have contributed to the loss of mitochondrial function (31, 32). We therefore incubated wild-type yeasts in RPMI medium with a sublethal concentration (10 mM) of H_2_O_2_ for 24 h and observed the emergence of small colonies at a low frequency, which did not grow in glycerol (data not shown).

These results indicate that the *petite* phenotype is adaptive within macrophages at early time points, but not at later times, probably due to the long period of starvation in the phagosome that prevents it from replicating. In agreement with this presumable advantage, the *petite* phenotypes arise from the respiratory-competent yeasts after 3 h to 1 day of phagocytosis, the same time period in which *Cgmip1*Δ shows a higher fitness. Importantly, these macrophage-induced *petites* can revert to a respiratory metabolism when grown again in the absence of stress.

### The *petite* phenotype triggered by fluconazole increases survival of phagocytosis at early time points.

**FIG 4:**
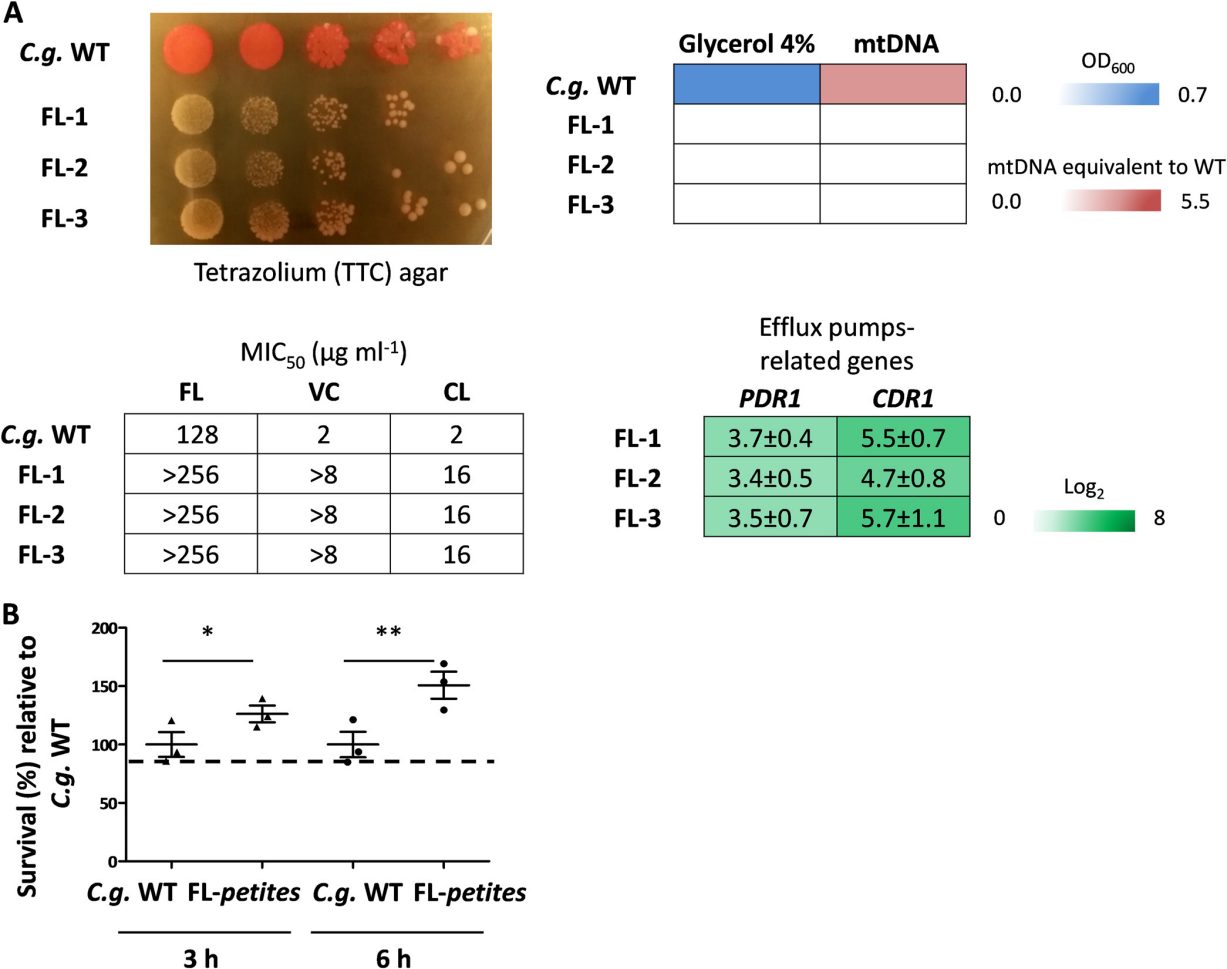
The *petite* phenotype triggered by fluconazole increases survival of phagocytosis at early time points. (A) Fluconazole-induced *petites* show the *petite* phenotype similar to *Cgmip1*Δ—small colonies and lack of mitochondrial reductive power, lack of growth in alternative carbon sources, absence of mitochondrial DNA (mtDNA), high resistance to azoles (all *n* = 3 with mean values or representative picture shown), and overexpression of efflux pump-related genes (mean ± SD, *n* = 3 independent experiments with 3 technical replicates each). FL, fluconazole; VC, voriconazole; CL, clotrimazole. (B) Fluconazole-induced *petites* (FL-1 to FL-3) show better survival of phagocytosis at early time points (mean ± SD, *n* = 3 with 1 donor in 3 independent experiments; each point represents a mean of 3 different colonies per donor, and each colony has 3 technical replicates). Statistically significantly different values (unpaired, two-sided Student’s *t* test on log-transformed ratios) are indicated by asterisks as follows: *, *P* ≤ 0.05; **, *P* ≤ 0.01. *C.g.*, C. glabrata.

It is known that azoles trigger (often temporary) mitochondrial dysfunction in C. glabrata (21, 33), which leads to fluconazole resistance through the upregulation of the efflux pump genes *CDR1* and *CDR2*, with the former being especially important in this resistance (16–18). In fact, *petite* mutants have very occasionally been isolated from patients undergoing fluconazole treatment (19, 22). Since our results showed an advantage of the genetically created *petite* strains after phagocytosis, we wondered whether fluconazole-induced *petites* share the same increased fitness. We therefore incubated wild-type yeasts for 8 h in RPMI medium with 8 μg/ml of fluconazole, half the concentration of the reported MIC_50_ for C. glabrata (34). Again, we observed the appearance of small colonies with a reversible and stable *petite* phenotype (Fig. 4A). When the stable strains were coincubated with macrophages for three and 6 h, all fluconazole-induced *petites* showed better survival in macrophages at both time points (Fig. 4B).

These results indicate a cross-resistance of the *petite* phenotype induced by and also protecting from both phagocytosis and fluconazole; exposure to fluconazole triggers a higher fitness of C. glabrata inside macrophages, and vice versa, fluconazole-resistant yeasts appear within macrophages.

### *Cgmip1*Δ shows higher basal expression of stress response-related genes and grows better under ER stress.

**FIG 5:**
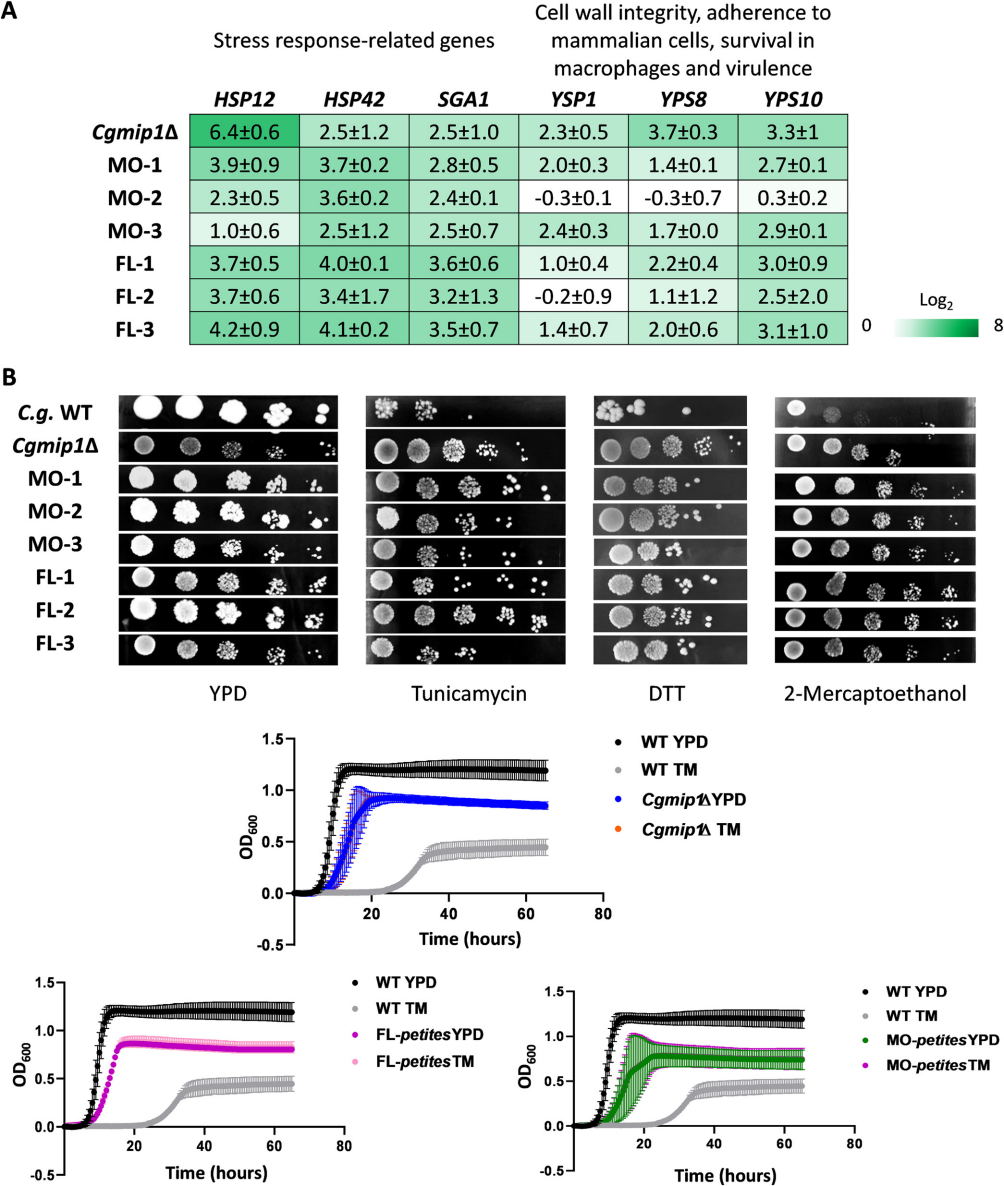
*Cgmip1*Δ shows higher basal expression of stress response-related genes and grows better under ER stress. (A) *Petite* variants of C. glabrata show high constitutive expression of stress-response genes even under nonstressed conditions (YPD) (mean ± SD, *n* = 3 independent experiments with 3 technical replicates each), and (B) exhibit better growth than their wild type under different ER stresses on plates as well as with tunicamycin (TM) in liquid cultures (mean ± SD, *n* = 3 independent experiments or representative picture shown); FL-1 to FL-3, fluconazole-induced *petites*; MO-1 to MO-3 macrophage-derived *petites*; *C.g.*, C. glabrata.

To understand why switching to a *petite* phenotype increases intraphagosomal fitness, we measured macrophage ROS production and damage, as it is known that C. glabrata is able to inhibit or detoxify ROS within the phagosome and also to burst the immune cell (9, 27). However, we did not observe significant differences between the wild type and *petite* for these parameters (Fig. S1). Since a reduced stress resistance correlates with a reduced intracellular survival (27), we also measured the basal and induced expression of different stress-response genes and tested the resistance to infection-related stresses. *Cgmip1*Δ and the macrophage- and fluconazole-derived *petite* strains showed an increased basal expression of a range of environmental stress response genes (*HSP12*, *HSP42*, and *SGA1*) and cell wall stress-related genes encoding yapsins (*YPS1*, *YPS10*, and *YPS8*) (Fig. 5A). These genes have been shown to be highly upregulated in the *petite* isolate BPY41 and may be involved in its hypervirulence (23). The yapsin *YPS* gene family has been implicated in the survival inside macrophages (35), and we found that while the three *YPS* genes were upregulated in C. glabrata, in the S. cerevisiae *petite* mutant (which does not survive phagocytosis better than its wild type) the most similar yapsin genes (*YPS1* and *MKC7*) showed no increased basal transcript levels (Fig. S2A).

10.1128/mBio.01128-21.1 FIG S1 *Petite* does not better inhibit ROS production by the hMDMs or increase damage compared to the wild type. (A) *Cgmip1*Δ shows similar ROS production to the wild type (PMA, phorbol myristate acetate) (*n* = 3, 3 independent experiments with 1 donor each and 3 technical replicates). (B) *Cgmip1*Δ shows a wild-type-like level of damage after 24 h of infection (*n* = 3, 3 independent experiments and 3 technical replicates). Download FIG S1, TIF file, 0.6 MB. Copyright © 2021 Siscar-Lewin et al. 2021 Siscar-Lewin et al. https://creativecommons.org/licenses/by/4.0/ This content is distributed under the terms of the Creative Commons Attribution 4.0 International license.

10.1128/mBio.01128-21.2 FIG S2 *YPS* genes and a *PDR1* pathway seem to be involved in the high survival rate to phagocytosis and ER stress resistance of the *petite* phenotype. (A) (Top) *Scmip1*Δ does not show high constitutive expression of its orthologues of the *YPS* genes (*YPS1* and *MKC7* orthologue to *YPS8* in C. glabrata) (mean ± SD, *n* = 3 independent experiments with 3 technical replicates each). (Bottom) *Scmip1*Δ grows similarly to the wild type on different ER stressors (representative picture shown). (B) *Cgmip1*Δ shows a wild-type-like level of resistance to oxidative stress (H_2_O_2_) on agar plates (representative picture shown). (C) The Pdr1 pathway seems to contribute to ER stress resistance but is not solely responsible (mean ± SD, *n* = 3 independent experiments with 3 technical replicates each). (D) Survival to phagocytosis by *pdr1*Δ single mutant and *Cgmip1*Δ+*pdr1*Δ double mutant (mean ± SD, *n* = 6 with 2 different donors in 3 independent experiments; each point represents the mean of 3 technical replicates). Statistically significantly different values (panel C, one-way ANOVA and Tukey’s test; panel D, unpaired two-sided Student’s t-test on log-transformed ratios) are indicated by asterisks as follows: *, *P* ≤ 0.05; **, *P* ≤ 0.01; ***, *P* ≤ 0.001. Download FIG S2, TIF file, 1.4 MB. Copyright © 2021 Siscar-Lewin et al. 2021 Siscar-Lewin et al. https://creativecommons.org/licenses/by/4.0/ This content is distributed under the terms of the Creative Commons Attribution 4.0 International license.

10.1128/mBio.01128-21.3 **FIG S3A to C** The *Cgmip1Δ petite* phenotype is found in clinical strains. (A) The clinical *petite* variants show formation of small colonies and lack mitochondrial reductive power (representative picture shown). (B) None of them show growth in alternative carbon sources and mostly show an absence of mitochondrial DNA (mtDNA) (mean of *n* = 3 independent experiments). (C) Most clinical isolates were able to grow (+) at the increased azole levels that indicated the resistance of the *petite* strains, which were generated *in vitro*. FL, fluconazole; VC, voriconazole; CL, clotrimazole FIG S2, TIF file, 1.4 MB. Copyright © 2021 Siscar-Lewin et al. 2021 Siscar-Lewin et al. https://creativecommons.org/licenses/by/4.0/ This content is distributed under the terms of the Creative Commons Attribution 4.0 International license.

10.1128/mBio.01128-21.4 **FIG S3D and E** The *Cgmip1Δ petite* phenotype is found in clinical strains. (D) Clinical isolates with the *petite* phenotype show increased survival to phagocytosis after 6 h (mean ± SD, *n* = 4 with 2 different donors in 2 independent experiments; each point represents a single survival test). Statistically significantly different values (one-way ANOVA and Dunnett’s test on log-transformed ratios) are indicated by asterisks as follows: *, *P* ≤ 0.05; **, *P* ≤ 0.01; ***, *P* ≤ 0.001. (E) *Petite* clinical strains generally grow better under ER stress than the ATCC 2001 reference strain (representative picture shown) FIG S2, TIF file, 1.4 MB. Copyright © 2021 Siscar-Lewin et al. 2021 Siscar-Lewin et al. https://creativecommons.org/licenses/by/4.0/ This content is distributed under the terms of the Creative Commons Attribution 4.0 International license.

We then tested *Cgmip1*Δ in different *in vitro* stress conditions, such as osmotic stress, metal stress, ER stress, oxidative stress, and cell wall stress, and found no relevant phenotype. Interestingly, *Cgmip1*Δ showed oxidative stress resistance comparable to the wild type (Fig. S2B), in contrast to mitochondrial mutants of S. cerevisiae that are known to be especially sensitive to H_2_O_2_ (36). Therefore, we discarded a decreased sensitivity to oxidative stress as a possible explanation for the higher survival of *Cgmip1Δ* within macrophages. However, *Cgmip1*Δ and the azole- and macrophage-induced C. glabrata *petites* showed better growth than their wild type under ER stress conditions (Fig. 5B), in contrast to the *petite* mutant of S. cerevisiae Sc*mip1*Δ (Fig. S2A). Since efflux pumps play a central role in azole resistance, we analyzed mutants lacking their main transcriptional activator Pdr1 (37, 38) in both the wild-type (*pdr1*Δ) and the *Cgmip1*Δ (*Cgmip1*Δ*+pdr1*Δ) background. As expected, both mutants grew poorly in increasing concentrations of fluconazole (Fig. S2C). However, under ER stress, the double mutant (lacking *CgMIP1* and *CgPDR1*) still exhibited significantly better growth than the single mutant and the wild type (Fig. S2C), showing that the *Cgmip1*Δ resistance to ER stressors cannot be solely dependent upon Pdr1-regulated pathways or functions. Evidently, additional resistance mechanisms exist in the *petite* phenotype. In agreement with these results, the double mutant *Cgmip1*Δ*+pdr1*Δ showed significantly higher survival than the wild type after phagocytosis by hMDMs, whereas a *pdr1*Δ single mutant was actually killed more (Fig. S2D). Thus, the Pdr1 pathway seems to be partially involved in stress resistance and macrophage survival of these strains, but this cannot be the sole underlying mechanism.

Overall, these results show that *petite* strains possess a constitutively active detoxifying response that, together with the overexpression of efflux pumps, confers ER stress resistance (in addition to azole resistance) and could explain the higher fitness within the phagosome observed at early time points.

### The *Cgmip1*Δ *petite* phenotype might be adaptive under infection-like conditions but not in commensalism.

**FIG 6:**
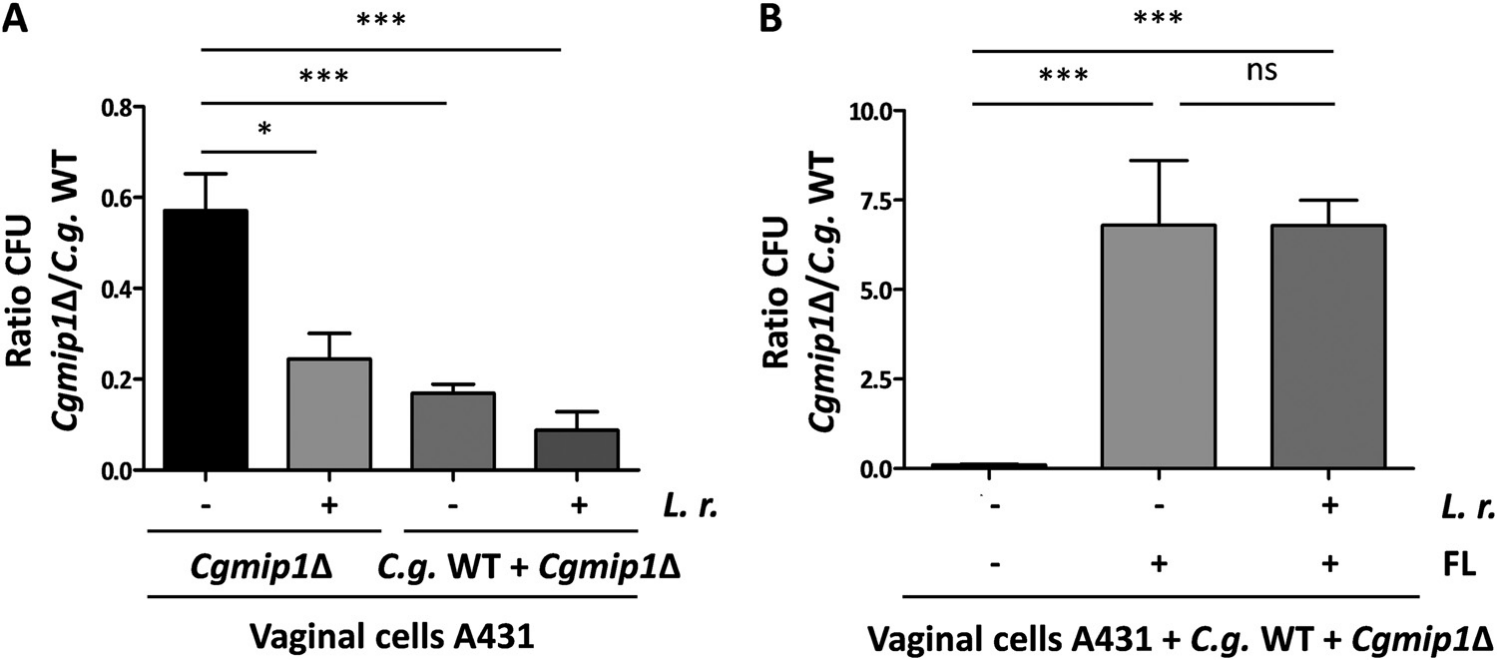
The *Cgmip1*Δ *petite* phenotype seems to be adaptive under infection-like conditions but not in commensal-like conditions. (A) The ratio of recovered *CgMIP1*-deleted to wild-type C. glabrata cells was low when they were incubated separately on vaginal cells (*Cgmip1*Δ/–). The presence of Lactobacillus rhamnosus (*L. r.*) further shifts that ratio toward a lower recovery of *Cgmip1*Δ, and these effects are exacerbated in a direct competition in the same wells (C. glabrata [*C. g.*] WT + *Cgmip*Δ). Data shown are the mean ± SD; *n* = 3 independent experiments. (B) In an infection model in the presence of fluconazole (FL), the effect is inverted. Without fluconazole, *Cgmip*Δ is outcompeted as before (note the scale), but it has a decisive advantage in the presence of the antifungal drug, independent of the commensal bacteria (mean ± SD, *n* = 3 independent experiments). Statistically significantly different values (unpaired, two-sided Student’s *t* test on log-transformed ratios) are indicated by asterisks as follows: *, *P* ≤ 0.05; ***, *P* ≤ 0.001.

We propose that the generally reduced growth of *petite* mutants will be disadvantageous when competing with other microorganisms in a commensal environment, such as the human gut or vagina. Therefore, we wondered whether the emergence of *petite* phenotype may only be adaptive under infection-like conditions, such as antifungal treatment with azoles. To test this, we incubated separately or simultaneously the wild type and *Cgmip1*Δ in the presence of vaginal cells and Lactobacillus rhamnosus for 24 h, mimicking a commensal-like situation (39), in either the absence or the presence of 8 μg/ml fluconazole. This fluconazole level is three times the concentration reported in vaginal fluids after a single oral dose (40), but a concentration half lower than the MIC_50_ for C. glabrata (34). As expected, we observed only 60% of the wild-type CFU for the *petite* strain when it was incubated alone with human epithelial cells and without antifungals after 24 h (Fig. 6A). The relative growth of *Cgmip1*Δ was further reduced in the presence of lactobacilli, and even more when coincubated with the wild type and bacteria (Fig. 6A). However, the *petite* strain massively out-competed the wild type when fluconazole was present (Fig. 6B). Surprisingly, we did not observe a reduction in the relative *Cgmip1*Δ CFU in the presence of both lactobacilli and fluconazole, as it was seen when both species grew together in the absence of the drug. The increased resistance to fluconazole of *Cgmip1*Δ may provide a better fitness in an acidic environment since it is known that fluconazole and acidic conditions created by *Lactobacillus* spp. have a synergistic activity. Indeed, fluconazole is known to be fungicidal in the presence of lactic acid for C. albicans (41) and acetate for C. albicans and C. glabrata (42).

In conclusion, these results show how in commensal-like and nontreated conditions, *Cgmip1*Δ is outcompeted by respiratory-competent yeast cells and is also less able to compete with commensal bacteria. We conclude that the *petite* phenotype likely emerges only under conditions where it is advantageous and then exists only transiently. These conditions include fluconazole treatment, but also uptake by macrophages.

### The *Cgmip1*Δ *petite* phenotype is found in clinical strains.

Our data so far indicate that the *petite* phenotype should only appear transiently or at low rates in patients but then provide significant advantages by increasing resistance to both phagocytes and antifungals. We therefore screened two collections of 146 clinical C. glabrata isolates in total, provided by two different laboratories. For sample collection, the incubation time was specifically extended to detect slower-growing yeasts. Sixteen strains were identified as *petite*; i.e., they showed a small colony size, absence of mitochondrial reductive power (Fig. S3A), and no growth in alternative carbon sources (Fig. S3B). The only common clinical characteristic these strains show is that the majority of them were isolated from patients with other underlying diseases (Table S1). Importantly, many of the *petites* were isolated from patients not currently undergoing antifungal therapy, and none were under azole treatment (Table S1). These strains showed the absence (likely rho^0^) and sometimes increased amounts of the mtDNA (rho^–^) fragment we screened for (Fig. S3B), without any visible difference in the phenotypes we tested for. Furthermore, like our experimentally created *petites* and independent of the amounts of mtDNA detected, clinical *petites* exhibited high resistance to azoles (Fig. S3C), although they had not been exposed to azole treatment (Table S1), and generally showed a higher survival inside macrophages at early time points compared to the wild type (Fig. S3D). Lastly, they grew better under ER stress than the wild type (Fig. S3E). These results indicate that the *petite* mutant can emerge during C. glabrata infections *in vivo* in clinical settings and that these exhibit all the resistances and characteristics we found in the experimentally created *petite* strains.

10.1128/mBio.01128-21.5 TABLE S1 Information about *petite* clinical strains of C. glabrata obtained from The Institute for Hygiene and Microbiology, Julius-Maximilians-University Table S1, DOCX file, 0.04 MB. Copyright © 2021 Siscar-Lewin et al. 2021 Siscar-Lewin et al. https://creativecommons.org/licenses/by/4.0/ This content is distributed under the terms of the Creative Commons Attribution 4.0 International license.

### *CgMIP1* sequencing of *petite* clinical isolates.

**FIG 7:**
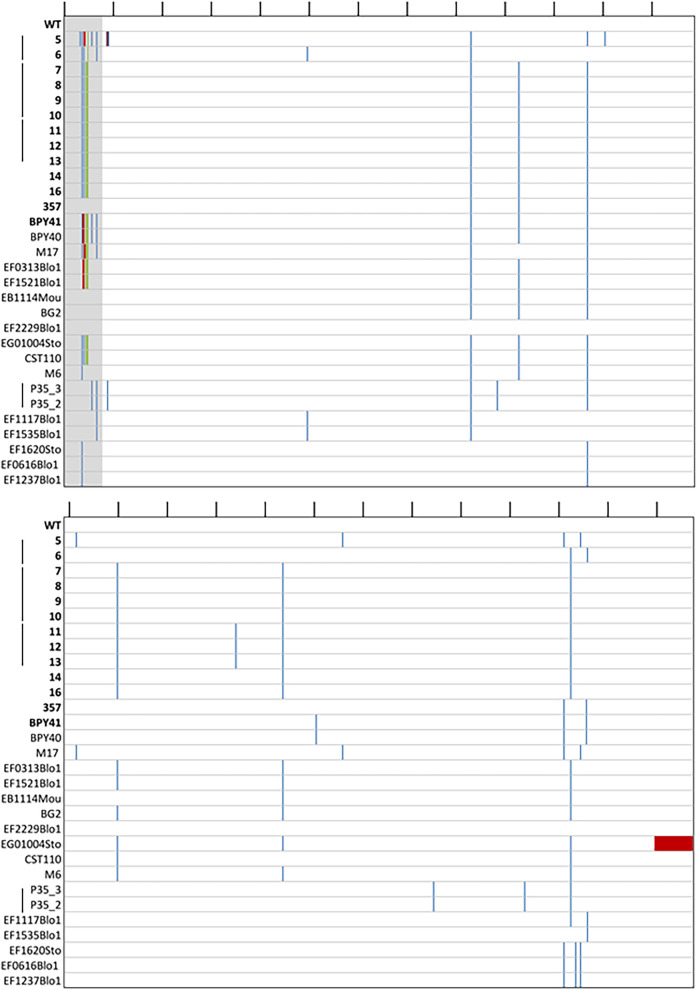
Mutation on the protein sequence of *CgMIP1* of C. glabrata clinical strains compared to the wild type. Wild type (ATCC 2001, WT); the first 14 strains marked in bold are *petite* mutants, and below there are the 17 respiratory-competent strains. Gray, predicted mitochondrial signal peptide of the wild type; blue, amino acid substitutions; green, insertions; red, deletions. The black lines on top indicate the amino acid position every 50 amino acids. The black lines next to the strain names indicate that those strains have been isolated from the same patient.

The fact that the mitochondrial polymerase gene *CgMIP1* shows a high value of positive selection (*d_N_/d_S_* = 3.40) during the diversification of C. glabrata as a species and the presence of *petite* strains in clinical isolates of C. glabrata led us to hypothesize that these two phenomena are connected. We therefore searched for mutations of *CgMIP1* by obtaining the genome sequences of 14 clinical strains isolated from 7 different patients, of which 13 were *petite* and 1 was respiratory competent. In comparison, we used the reference C. glabrata strain ATCC 2001 and 16 respiratory-competent clinical strains, whose genome sequences have been previously obtained (43). Compared to the wild-type strain, we found different mutations along the *MIP1* gene sequence, but we did not observe a specific common mutation pattern for the *petite* strains (Fig. 7). Furthermore, none of these mutations were found in the predicted polymerase or exonuclease domains, which are important for the function of the protein (44). Interestingly, we found variations of the N-terminal mitochondrial targeting sequence, which we determined by TargetP 2.0. Twelve *petites* (98.3%) show insertions of up to three more amino acids in the positions 24S, 25M, and 26L/R, in comparison to the respiratory-competent strains, from which only six contained such insertions (35.3%). Furthermore, we looked for similar mutations in other proteins with known or expected mitochondrial localization. Dss1 is an exonuclease of the mitochondrial degradasome, and *CAGL0K03047g* is an ortholog of the S. cerevisiae *ABF2* gene, which has a role in mtDNA replication. Like Mip1, both are essential for mitochondrial biogenesis (45) and maintenance (46). Hem1 is localized in the mitochondrial matrix and required for heme biosynthesis in S. cerevisiae. Pgs1 is a mitochondrially localized protein whose deletion leads to increased azole resistance (26), and Pup1 is a mitochondrial protein that is upregulated by Pdr1 in azole-resistant strains (23). We did not detect variability similar to Mip1’s in any of these investigated protein sequences, independent of whether a well-defined mitochondrial transfer peptide was detectable (Abf2, Hem1) or not (Dss1, Pgs1, Pup1).

## DISCUSSION

Despite the fact that C. glabrata is phylogenetically more closely related to the baker’s yeast S. cerevisiae than to the pathogenic C. albicans, it has by now become the most important non-Candida albicans *Candida* (NCAC) species to cause disease and is of growing concern in clinics (47). The relatively low host cell damage and immune responses that C. glabrata elicits, combined with its ability to survive and replicate within macrophages, indicates that stealth and persistence are its main virulence strategies during infection (4). However, residing for a long time within the human body requires phenotypic plasticity to survive changing stresses, such as osmotic, ER, and oxidative stress, hypoxia, and starvation (48–53). One mechanism that leads to the better host adaptation and virulence in human-pathogenic bacteria and fungi is the inactivation or loss of specific genes, which are then known as antivirulence genes (10, 11).

Specifically, in C. albicans and C. glabrata, mutations decreasing mitochondrial function can affect host-pathogen interactions. Although C. albicans is considered a *petite*-negative species (13), a mutant with uncoupled oxidative phosphorylation was recovered after serial passaging of wild-type C. albicans through murine spleens (54). It showed higher tolerance to ROS, altered cell wall composition, resistance to phagocytosis by neutrophils and macrophages, as well as an increased persistence and higher fungal burden in mice. Of note, in contrast to its progenitor, this strain did not kill infected mice any more. The authors pointed out that the uncoupled oxidative phosphorylation lowers intrinsic ROS production, which may be advantageous inside the phagosome, while the altered cell wall composition may diminish immune recognition. The mutant also showed a lower susceptibility to fluconazole and voriconazole due to increased expression of the efflux pump-encoding gene *MDR1* (55). In another example, a respiratory-competent C. glabrata strain and its *petite* mutant were sequentially isolated from a patient undergoing long-term fluconazole treatment (22, 23). The mutant showed a high expression of virulence- and efflux pump-related genes, oxido-reductive metabolism and stress response genes, as well as cell wall-related genes. It also led to a higher mortality in neutropenic mice and a higher tissue burden in immunocompetent mice (23), and it was also resistant to azoles due to the *PDR1*-dependent upregulation of the efflux pump-encoding genes *CDR1* and *CDR2*. The mitochondrion-related gene, *PUP1*, was also strongly upregulated in a *PDR1*-dependent manner. Interestingly, enhanced virulence of C. glabrata associated with high upregulation of *CDR1* and *PUP1* has been observed in azole resistance clinical isolates resulting from gain of function mutations (GOF) in the *PDR1* gene (23). It was therefore speculated that both genes may contribute to favor C. glabrata in host interactions, in a still unknown manner. Thus, the *petite* phenotype may constitute a relevant pathogenic form of C. glabrata, and genes involved in mitochondrial function may be considered potential antivirulence genes (11).

In agreement with these previous findings, our study highlights the adaptive advantage that the lack of mitochondrial function has for C. glabrata under infection conditions. Likely not coincidentally, the mtDNA polymerase-encoding gene *CgMIP1* seems to have been under selective pressures during the evolution of C. glabrata (5). We found that deletion of this gene leads to loss of mtDNA and triggers the *petite* phenotype in both C. glabrata and S. cerevisiae. This phenotype confers a survival advantage at early time points after phagocytosis, but only for C. glabrata and not for S. cerevisiae. In addition, *petite* variants appeared from phagocytosed wild-type cells at an appreciable frequency, especially at early time points during their interaction with macrophages. We argue that these were likely induced by the intraphagosomal environment and provided an immediate advantage to the fungus. However, within the phagosome, glucose is absent, and only alternative carbon sources are available (carboxylic acids, amino acids, peptides, *N*-acetylglucosamine, and fatty acids), which require mitochondrial oxido-reductive power for their metabolism (29, 30). We assume that this is the reason that in the long term, after 4 and 7 days, the advantage of the *petite* phenotype is reverted, as they starve and are recovered in ever lower numbers. Of note, the fact that in this experimental model oxygen is present at normal atmospheric levels may confer an advantage to the respiratory-competent wild type. In infected tissue *in vivo*, in contrast, oxygen levels are low, and it is known that hypoxia modulates the innate immune response and enhances phagocytosis (56, 57). In this case, the *petite* phenotype may even confer an adaptive advantage over respiratory-competent yeasts, which must rewire their metabolism upon confrontation with phagocytes. In addition, we observed that the *petite* phenotype can reverse if the fungi find themselves outside macrophages and the associated stresses. It seems feasible therefore that macrophage-induced *petites* may regain their normal growth behavior *in vivo* if the fungus escapes the phagocytes and the change into a *petite* phenotype represents a temporary adaptation of C. glabrata to adverse conditions.

Importantly, in addition to reversible *petites*, we collected several nonreversible macrophage-derived *petite* variants that allowed us to study their phenotype. They showed high resistance to azoles, in addition to other typical *petite* characteristics. In turn, stable fluconazole-derived *petites* showed the expected loss of susceptibility to azoles and, surprisingly, a better survival to phagocytosis as well. It is known that fluconazole can trigger (temporary) loss of mitochondrial function in C. glabrata and S. cerevisiae and, as a consequence, an increased fluconazole resistance (16–18, 21). However, to our knowledge this study shows for the first time that phagocytosis and intraphagosomal residence can lead to the emergence of fluconazole resistance, and vice versa, in a potentially clinically important cross-resistance phenomenon.

The mechanistic basis of how these resistances develop is not completely understood: in contrast to our collection of clinical *petites*, the clinical *petite* variants reported so far have all been isolated from patients undergoing fluconazole treatment (19, 22), and most of the *petite* isolates analyzed here lack mtDNA, but synthesis inhibition or degradation of mtDNA by the action of azoles has not yet been reported. In fact, it was shown that azole exposure does not always lead to a loss of mtDNA, but rather, damages mitochondrial components (17, 18, 21). Fluconazole-induced *petite* can revert at a frequency of 1.5 × 10^−2^ (21), which suggests a genetic or epigenetic regulation. Our data indicate that C. glabrata turns *petite* within the macrophages due to phagosomal oxidative stress, since we observed similar conversions during incubation with H_2_O_2_. It is known that oxidative stress can trigger mitochondrial damage and loss of activity by affecting mitochondrial membrane permeability, the respiratory chain, or the mtDNA (31, 32). These factors could be at work during the induction of the *petite* phenotype in macrophages. Importantly, like azole-induced *petites*, we found that a fraction of these macrophage-derived *petite* phenotypes were reversible.

The loss of mitochondrial function also activates compensatory pathways, such as detoxifying mechanisms and cell wall remodeling (33). Indeed, all our *petite* strains showed constitutive expression of the efflux pump-related (and azole resistance-mediating) genes *PDR1* and *CDR1*, and some transcription of heat shock protein-encoding genes such as *HSP12* and *HS42* and *SGA1* (23, 58). Moreover, all C. glabrata *petites* exhibited a surprisingly high resistance to ER stressors, which was not found in S. cerevisiae. We suggest that this ER stress resistance is induced by mitochondrial dysfunction, possibly by way of the interorganelle tether ERMES (endoplasmic reticulum and mitochondrial encounter structures). Interestingly, so far, only the opposite direction has been observed, where ER stress leads to mitochondrial dysfunction (59). Our data may therefore hint toward a bidirectional signaling between the two organelles. This response also seems to confer an adaptive advantage in the phagosome, since it has been shown that ROS may act not alone in killing the fungus but in combination with additional stresses; suppression of ROS by macrophages alone does not increase the fungal survival (6). In addition, this ER stress resistance would also confer protection against the generic ER stress that pathogens face during infection (35, 60, 61). We also showed that the *PDR1* pathway is at least partially involved in the ER stress resistance as well as in macrophage survival, in agreement with previous findings (23). The majority of *petites* also showed increased expression of members of the *YPS* gene family. It has been suggested that Yps-mediated cell wall remodeling can play a role in altering or suppressing macrophage activation (35), and we hypothesize that this may contribute to the better survival of *petite* variants within the phagosome. However, the exact mechanisms for the increased survival of *petite* remains unknown, not the least because the general basis for C. glabrata macrophage survival is still largely unknown (6). Of note, S. cerevisiae, which did not benefit from a *petite* phenotype in macrophages, also did not show constitutive *YPS* orthologue expression or ER stress resistance. Higher oxidative stress resistance does not seem to play a role, as *Cgmip1*Δ grew similarly to the wild type under oxidative stress. In contrast, different mitochondrial mutants of S. cerevisiae even show increased sensitivity to oxidative stress (36). The altered cell wall, and especially the increased mannan exposure of *Cgmip1*Δ, can explain the increased phagocytosis rate, as this is known to be mannan-dependent (25, 62), but also a better survival (27). Clearly, the induction of a *petite* phenotype by either azoles or phagocytosis had a strong influence on the macrophage-fungus interactions, but overall, benefits C. glabrata in the phagosome.

The *petite* phenotype shows, nonetheless, a strong handicap in fitness and competitiveness in our commensal-like model, likely due to its slow growth, as observed before (63). However, in our model of vaginal candidiasis treated with fluconazole, the *petite* phenotype shows a steady advantage. Therefore, we suggest that the *petite* phenotype, which also appears naturally and in the absence of stress at low frequencies, serves as a bet-hedging strategy to face stressful conditions, such as phagocytosis or azole exposure, in C. glabrata. Similar phenotypes are described in bacteria, for which it is known that microorganisms that give rise to heterogeneous populations and phenotypic switching are more likely to survive in fluctuating environments than otherwise “stable” populations (64, 65). Important intracellular pathogens such as Staphylococcus aureus and Salmonella are known to form small colony variants (SCVs), a phenotype analogous to *petite*, as part of the bacterial heterogeneity that might confer an adaptive advantage upon environmental changes (64, 66, 67). These show a decrease in antibiotic susceptibility and link to chronic and relapsing, often therapy-refractory, infections. Moreover, they show reduced expression of virulence factors and higher adhesion, which promotes internalization in host cells, and facilitate immune-evasion and long-term persistence within their hosts (68, 69). SCVs from many Gram-positive and -negative bacteria have been recovered from clinical tissues (68, 69). On the yeast counterpart, so far, only a *petite* mutant of C. glabrata has been reported to possess any pathogenic advantage (23). Analogous to the SCVs, these mutants have been isolated from cases of antifungal treatment (19) and recurrent fungemia (22), with a decreased susceptibility to antifungals and increased fungal burden in animal models of infection (23). Furthermore, recently, it has been shown that a negative correlation between fitness costs derived from drug resistance and virulence is not always the case, but in fact, virulence could be maintained or even increased in the presence of such costs that result in a reduced growth rate in C. glabrata (70). This agrees with our hypothesis of the *petite* phenotype as an advantageous strategy during infection despite its slower growth. The same slow growth within macrophages, however, also led to depletion of *petites* in our long-term experiments over a week, making it more difficult to imagine a permanent selection advantage of this phenotype in the human host. Two things, however, must be considered, first that the oxygen level (and with it the relative advantage of functional mitochondria) is lower within tissue than in cell culture or liquid medium growth experiments (71). Second, the *petites* may also escape macrophages before these effects come into play, either via an unknown, potentially nonlytic escape route or by bursting the macrophage (9, 72). A highly interesting possibility in this context is that the presence of cohabiting *petites* may benefit wild-type cells in the same macrophage. In Cryptococcus gattii, a spontaneous-appearing variant with tubular mitochondria is itself replication-deficient but resistant to phagosomal stresses (73). In coinfected macrophages, this seems to protect strains with normal mitochondria and increases their proliferation significantly. Whether a similar “division of labor,” as the authors call it (73), may allow *petites* to help wild-type C. glabrata (and potentially benefit themselves by having the wild type burst the macrophage due to their growth) is an interesting open question that we will address in the future.

Given the potential advantages of *petite* variants during infection, one would expect to frequently find C. glabrata *petite* phenotypes in clinical samples, but although *petites* have been reported (19, 22), these reports seem to be rare. Nonetheless, when we specifically looked for the *petite* phenotype in clinical isolates, we found 16 of these strains even in the absence of antifungal treatment. They shared all the hallmark features with *Cgmip1*Δ, macrophage-, and fluconazole-derived *petites*; the majority showed loss of mtDNA, azole resistance, increased survival upon phagocytosis, and increased ER stress profile, which suggests a common mode of resistance within the macrophages. It may well be that C. glabrata *petite* phenotypes—possibly triggered by interactions with macrophages—are actually more common in clinical samples but potentially overlooked because of their slow growth. Paralleling our finding of a host activity-induced increase in antifungal resistance, it has recently been shown that in diverse *Candida* species, the toxic metabolite methylglyoxal—associated among others with neutrophils—induces not only genes of its own catabolism but also genes for azole resistance (82). The authors suggest that host-derived methylglyoxal may thereby contribute to clinical resistance, not unlike our model for the macrophage-induced *petites*.

We started these investigations because of the signs of recent selection on the mitochondrial DNA polymerase gene *CgMIP1*. We hypothesized that this could indicate a role in the adaptation of C. glabrata to host environments. Indeed, some (but not all) of the *petite* clinical isolates we investigated showed mutations in the *CgMIP1* gene sequence, and the majority showed an insertion pattern in the presumable mitochondrial targeting sequence, which may relate to their *petite* phenotypes via a reduction of mitochondrial polymerase function. In addition, we found that other mitochondrion-related proteins did not show similar mutation frequency, which supports the idea that CgMip1 may still be a target for selection. Moreover, it has been shown that point mutations in the polymerase domain of the orthologous *MIP1* gene in S. cerevisiae can trigger emergence of *petites*, and this frequency increased with higher temperatures. Specifically, the mutation E900G yielded from 6% *petites* at 28°C to 92% at 36°C (74). While many of these *petites* were rho^0^—completely devoid of mtDNA—some still contained amplified mtDNA fragments that map to various positions of the mtDNA and were considered rho^−^ (74). These mtDNA fragments can rescue strains that are respiratory deficient due to mutations in mtDNA by recombination after crossing (75, 76). Interestingly, we observed mtDNA fragments in our sequence data of clinical *petite* strains (data not shown), similar to S. cerevisiae rho^−^. We cannot discard the possibility that mtDNA can be restored by recombination after crossing *in vivo*, since while it has not yet been observed, C. glabrata has most likely not lost its ability to mate (43, 77). In addition, we also found that the wild-type strain of C. glabrata and its two most closely related human pathogens, Nakaseomyces nivariensis and Nakaseomyces bracarensis, show the substitution E926D in Mip1 compared to the environmental species Nakaseomyces delphensis, Nakaseomyces bacillisporus, and Candida castelli. Since this is equivalent to the E900 position in S. cerevisiae, which upon mutation increases the frequency of *petite* occurrences, we hypothesize that, on the one hand, C. glabrata could more easily turn *petite* than environmental *Nakaseomyces* species, but on the other hand, it can also retain mtDNA in its genome as a possible mechanism to recover mitochondrial function once the *petite* phenotype is not adaptive. These tantalizing hypotheses will be tested in the near future.

Alternatively, the C. glabrata *CgMIP1* gene sequence may be the target of epigenetic regulation, as epigenetics have been suggested to be involved in the reversion of *petite* phenotype to wild type (21). In these models, mtDNA levels are reduced down to a (near) complete loss of mitochondrial function. Upon resumption of polymerase function, the mitochondrial function and growth then revert to normal. The ability to switch between *petite* and non-*petite* phenotype would confer C. glabrata an important phenotypic plasticity to adapt to the host’s changing environments; the *petite* phenotype may be less competitive as a commensal but much fitter in infection situations with active phagocytes and antifungal treatment.

In conclusion, this study shows how temporary mitochondrial dysfunction triggers a *petite* phenotype in C. glabrata under infection-like conditions, with the potential to confer cross-resistance between the macrophages and azole antifungal treatment. This has three implications. First, it adds mitochondrial function to the list of potential antivirulence factors, since its loss results in a gain in virulence potential in the form of stress resistance. Second, it has implications for the treatment of C. glabrata, as fluconazole may inadvertently increase the fitness of the fungus against the innate host defenses. Due to the *petite* morphology and long generation times, such resistant isolates may then be missed in standard diagnostics. Third, our observations provide a potential clinically relevant route for the emergence of azole resistance of C. glabrata by immune activities, a new paradigm in the development of antifungal resistance.

## MATERIALS AND METHODS

### Screening and acquisition of suspected petite C. glabrata strains.

In the course of routine diagnostics—executed by the Institute of Hygiene and Microbiology in Würzburg, Germany—all accumulated chromogenic *Candida* agar plates (CHROMagar; Becton, Dickinson, New Jersey, USA) were systematically collected and incubated for at least 7 days at 37°C prior to screening. After incubation, plates were visually screened concerning growth, color, and morphology. Only suspected C. glabrata small colony variants were subisolated and reincubated for a minimum of 3 days at 37°C. In case of a confirmed growth behavior, final species identification was executed by matrix-assisted laser desorption ionization–time of flight mass spectrometry MALDI-TOF) (bioMérieux, Paris, France).

From a total of 3,756 agar plates—originating from various clinical specimens examined between November 2019 and June 2020—466 exhibited growth after incubation. A total of 525 different strains were identified through chromogenic media, of which the majority (312) presented in a green color, suggesting C. albicans, whereas in 170 cases mauve colonies were observed. A total of 82 of these were identified as C. glabrata using MALDI-TOF. Based on morphology, 41 strains were suspected to be *petites*, which was finally confirmed in 20 cases.

### Strains and growth conditions.

All strains used in this study are listed in Table S2. The C. glabrata mutant strains are derivatives of the laboratory strain ATCC 2001. In each strain a single open reading frame (ORF) was replaced with a bar-coded NAT1 resistance cassette in the strain ATCC 2001. All yeast strains were routinely grown overnight in YPD (1% yeast extract, 1% peptone, 2% glucose) at 37°C in a 180-rpm shaking incubator.

10.1128/mBio.01128-21.6 TABLE S2 Strains used in this study Table S2, DOCX file, 0.02 MB. Copyright © 2021 Siscar-Lewin et al. 2021 Siscar-Lewin et al. https://creativecommons.org/licenses/by/4.0/ This content is distributed under the terms of the Creative Commons Attribution 4.0 International license.

To analyze sensitivity to H_2_O_2_ and ER stress, 5 μl of serial diluted yeast cultures (10^7^, 10^6^, 10^5^, 10^4^, 10^3^, and 10^2^ cells/ml) were dropped on solid YPD medium (YPD, agarose 2%) containing increasing concentrations of H_2_O_2_ (7.5 mM, 10 mM, and 12 mM), tunicamycin (2 μg/ml), DTT (10 mM) or 2-mercaptoethanol (12 mM). Pictures were taken after 48 h of incubation at 37°C, unless indicated otherwise.

Mitochondrial activity was visualized by growing serial dilutions of the strains on YPD agar containing 0.02% TTC (2,3,5-triphenyltetrazolium chloride) (Sigma-Aldrich) and incubating cells in minimum medium (1% yeast nitrogen base, 1% amino acids, 0.5% ammonium sulfate) with 4% glycerol as the sole carbon source for 4 days at 37°C in a 180-rpm shaking incubator.

### Growth assays.

To analyze stress sensitivity, 5 μl of a yeast cell suspension (2 × 10^7^ cells/ml) was added to 195 μl medium in a 96-well plate (tissue culture test plate; TPP Techno Plastic Products AG) containing liquid YPD or YPD and different concentrations of fluconazole (10, 25, 35, 50, 75, and 100 μg/ml) and tunicamycin (0.5, 1, 1.5, 2, and 3 μg/ml). For the ER stress analysis, yeasts were incubated in YPD and tunicamycin (1.5 μg/ml). The growth was monitored by measuring the absorbance at 600 nm every 30 min for 150 cycles at 37°C, using a Tecan reader (Infinite M200 PRO plate reader; Tecan Group GmbH) with orbital shaking. All experiments were done in independent biological triplicates on different days and are shown as the mean with standard deviation (SD) for each time point.

The MIC_50_ was determined in a 96-well plate (tissue culture test plate; TPP Techno Plastic Products AG) containing minimum medium (1% yeast nitrogen base, 1% amino acids, 0.5% ammonium sulfate, and 2% glucose) with increasing concentrations of fluconazole (FL) (4, 8, 16, 32, 64, 128, and 256 μg/ml), voriconazole (VC) (0.5, 1, 2, 4, 8, and 16 μg/ml), and clotrimazole (CL) (1, 2, 4, 8, and 16 μg/ml) at 37°C.

### Fungal RNA isolation.

For preparation of RNA from *in vitro*-cultured *Candida* cells, stationary-phase yeast cells were washed in phosphate-buffered saline (PBS) and the optical density (OD) was adjusted to 0.4 in 5 ml liquid YPD. After 3 h, cells were harvested and centrifuged. The isolation of the fungal RNA was performed as previously described (78). The RNA was then precipitated by adding 1 volume of isopropyl alcohol and one-tenth volume of sodium acetate (pH 5.5). The quantity of the RNA was determined using the NanoDrop spectrophotometer ND-1000 (NRW International GmbH).

### Expression analysis by reverse transcription-quantitative PCR (qRT-PCR).

The cDNA was synthesized from DNase-treated RNA (1,000 ng) using 0.5 μg oligo-dT_12-18_, 100 U Superscript III reverse transcriptase, and 20 U RNaseOUT recombinant RNase inhibitor (all from Thermo Fischer Scientific) in a total volume of 20 μl for 2 h at 42°C followed by heat inactivation for 15 min at 70°C. Quantitative PCR with EvaGreen QPCR mix II (Bio&SELL) was performed with 1:10 diluted cDNA. Primers were used at a final concentration of 500 nM. Target gene expression was calculated using the ΔΔ*CT* method (79), with normalization to the housekeeping genes *CgACT1* for C. glabrata or *ScACT1* for S. cerevisiae. For mtDNA quantification, yeast DNA was extracted following the Harju et al. protocol (80), and 100 ng was the reaction concentration. *ScCOX3* and *CgCOX3* were used as mitochondrial target genes and *CgACT1* or *ScACT1* as housekeeping genes. All experiments were done in independent biological triplicates on different days and are shown as the mean with standard deviation (SD) for each time point.

### Chitin, mannan, and β-glucan exposure.

To measure chitin content, yeasts from an overnight culture were washed in PBS and incubated with 9 ng/ml of wheat germ agglutinin (WGA)-FITC diluted in PBS for 1 h at room temperature. After washing with PBS, fluorescence was quantified by flow cytometry (BD FACS Verse; BD Biosciences, Franklin Lakes, NJ, USA) counting 10,000 events. For mannan quantification, yeast cells were washed with PBS and incubated with concanavalin A-647 for 30 min at 37°C. After washing with PBS, fluorescence was quantified also by flow cytometry. For β-glucan staining, yeast cells were washed with PBS and incubated with 2% bovine serum albumin (BSA) for 30 min at 37°C, followed by a first step of 1 h of incubation with a monoclonal anti-β-glucan antibody (Biosupplies) (diluted 1:400 in 2% BSA) and a second step of 1 h of incubation with an Alexa Fluor 488 conjugate secondary antibody (Molecular Probes) (diluted 1:1,000 in 2% BSA). Fluorescence was again quantified by flow cytometry. All experiments were done in independent biological triplicates on different days, and shown as the mean with standard deviation (SD) for each time point.

### Isolation and differentiation of human monocyte-derived macrophages (hMDMs).

Blood was obtained from healthy human volunteers with written informed consent according to the declaration of Helsinki. The blood donation protocol and use of blood for this study were approved by the Jena institutional ethics committee (Ethik-Kommission des Universitätsklinikums Jena, permission no. 2207-01/08). Human peripheral blood mononuclear cells (PBMCs) from buffy coats donated by healthy volunteers were separated through lymphocyte separation medium (Capricorn Scientific) in Leucosep tubes (Greiner Bio-One) by density centrifugation. Magnetically labeled CD14-positive monocytes were selected by automated cell sorting (autoMACs; MiltenyiBiotec). To differentiate PBMC into human monocyte-derived macrophages (hMDMs), 1.7 × 10^7^ cells were seeded into 175-cm^2^ cell culture flasks in RPMI 1640 medium with l-glutamine (Thermo Fisher Scientific) containing 10% heat-inactivated fetal bovine serum (FBS; Bio&SELL) and 50 ng/ml recombinant human macrophage colony-stimulating factor (M-CSF; ImmunoTools). Cells were incubated for 5 days at 37°C and 5% CO_2_ until the medium was exchanged. After another 2 days, adherent hMDMs were detached with 50 mM EDTA in PBS and seeded in 96-well plates (4 × 10^4^ hMDMs/well) for the survival assay, in 12-well-plates (4 × 10^5^ hMDMs/well) for the intracellular replication assay with 100 U/ml interferon-γ (IFN-γ), and in 24-well-plates for the long-term experiment (1.5 × 10^5^ hMDMs/well) without IFN-γ. Prior to macrophage infection, medium was exchanged with serum free-RPMI medium and 100 U/ml IFN-γ. For the long-term experiment, medium was exchanged with RPMI 1640 containing 10% human serum (Bio&Sell 1; B&S human serum sterilized AB male, lot BS.15472.5).

### Phagocytosis survival assay.

Mutant strains were washed in phosphate-buffered saline (PBS), and total numbers of cells were assessed by the use of a hemocytometer. MDMs in 96-well-plates were infected at a multiplicity of infection (MOI) of 1, and after 3 h and 6 h of coincubation at 37°C and 5% CO_2_, non-cell-associated yeasts were removed by washing with RPMI 1640. To measure yeast survival in MDMs, lysates of infected MDMs were plated on YPD plates to determine CFU.

The long-term experiment was performed in 24-well-plates where the cells were infected with an MOI of 1 and incubated for 1 week at 37°C and 5% CO_2_. After 3 h of coincubation, cells were washed with PBS, and medium was exchanged with RPMI 1640 containing 10% human serum. At the 3 h time point, both supernatant and lysate were plated. Until the 1-day and 7-day times points, one-third of the medium was exchanged every day with RPMI 1640 with 10% human serum. Then, only the lysate was plated. The lysate of 4 different wells was diluted accordingly, and 200 CFU were plated on 4 YPD agar plates, which were then incubated for 48 h at 37°C. The frequency of *petites* was determined via small colonies that were unable to grow with 4% glycerol as the sole carbon source in minimal medium (1% yeast nitrogen base, 1% amino acids, 0.5% ammonium sulfate). The growth was assayed for 3 days at 37°C in a 180-rpm shaking incubator. The frequency of spontaneous *petites* was calculated by incubation of 1.5 × 10^5^ cells ml^−1^ in RPMI 1640 for 7 days. At 3 h, 1 day, 4 days, and 7 days samples were collected and diluted, and 200 CFU were plated on 6 YPD agar plates, which were incubated for 48 h at 37°C.

### Replication within hMDMs.

To quantify yeast intracellular replication, C. glabrata cells were labeled with 0.2 mg/ml fluorescein isothiocyanate (FITC) (Sigma-Aldrich) in carbonate buffer (0.15 M NaCl, 0.1 M Na_2_CO_3_, pH 9.0) for 30 min at 37°C. Then, yeast cells were washed in PBS, and macrophages were infected at an MOI 5 for 6 h. Afterward, macrophages were washed with PBS and lysed with 0.5% Triton-X-100 for 15 min. Released yeast cells were washed with PBS, with 2% BSA in PBS, and counterstained with 50 μg/ml Alexa Fluor 647-conjugated concanavalin A (ConA) (Molecular Probes) in PBS at 37°C for 30 min. The ConA-AF647-stained yeast cells were washed with PBS and fixed with Histofix (Roth) for 15 min at 37°C. As FITC is not transferred to daughter cells, differentiation of mother and daughter cells was possible; the ratio of FITC-positive and -negative yeast cells was evaluated by flow cytometry (BD FACS Verse; BD Biosciences, Franklin Lakes, NJ, USA), counting 10,000 events. Data analysis was performed using the FlowJo 10.2 software (FlowJo LLC, Ashland, OR, USA). The gating strategy was based on the detection of single and ConA-positive cells and exclusion of cellular debris.

For quantification of intracellular replication by fluorescence microscopy, cells were fixed with Histofix (Roth) after incubation with macrophages and stained for 30 min at 37°C with 25 μg/ml ConA-AF647 (Molecular Probes) to visualize nonphagocytosed yeast cells. Then they were mounted cell side down in ProLong Gold antifade reagent (Molecular Probes). As FITC is not transferred to daughter cells, differentiation of mother and daughter cells was possible, and intracellular replication was observed by fluorescence microscopy (Leica DM5500B and Leica DFC360).

### Detection of ROS in hMDMs.

ROS production was measured by luminol-enhanced chemiluminescence quantification, and all cells and reagents were prepared in RPMI 1640 without phenol red. MDMs were grown in white 96-well plates in 100 μl medium per well. Overnight yeast cultures were washed in RPMI 1640 and counted, and 50 μl was added to hMDMs (giving an MOI of 10). For control experiments, hMDMs were left untreated in 150 μl RPMI 1640, or 100 nM phorbol myristate actetate (PMA) (Sigma-Aldrich) was added to 50 μl RPMI 1640. All samples were prepared in triplicate. Prior to quantification, 50 μl of a mixture containing 200 μM luminol and 16 U horseradish peroxidase in RPMI 1640 was immediately added. Luminescence was measured every 3 min over a 3-h period of incubation at 37°C using a microplate reader (Tecan Infinite 200).

### Macrophage damage assay.

Macrophages were infected with *Candida* cells as described above and incubated for 24 h. Release of the cytoplasmic enzyme lactate dehydrogenase (LDH) was measured as a marker for necrotic cell damage using a cytotoxicity detection kit (Roche) according to the manufacturer’s instructions. The background LDH value of uninfected macrophages (hMDMs) was subtracted, and the corrected LDH release was expressed as the percentage of high (full lysis) control (maximum LDH release induced by the addition of 0.25% Triton X-100 to uninfected macrophages for 5 min).

### Competition assay.

This experiment was adapted from Graf et al. (39). A431 vaginal epithelial cells (Deutsche Sammlung von Mikroorganismen und Zellkulturen [DSMZ] no. ACC 91) were routinely cultivated in RPMI 1640 medium with l-glutamine (Thermo Fisher Scientific) containing 10% heat-inactivated fetal bovine serum (FBS; Bio&SELL) at 37°C and 5% CO_2_ for no longer than 15 passages. For detachment, cells were treated with Accutase (Gibco, Thermo Fisher Scientific). For use in experiments, the cell numbers were determined using a Neubauer chamber system and seeded in a 6-well-plate (4 × 10^5^ cells/well) for 3 days. For infection experiments, the medium was exchanged with fresh RPMI 1640 without FBS. L. rhamnosus (ATCC 7469) was grown in MRS broth for 72 h at 37°C. Before infection, bacteria were harvested, washed with PBS, and adjusted to an optical density at 600 nm (OD_600_) of 0.2 (∼1 × 108 CFU/ml) in RPMI 1640. Then, one-third of the total volume of the well of a 6-well-plate was inoculated for 18 h prior to infection with C. glabrata. These wells were colonized with C. glabrata wild type and mutant separated or mixed in equal cell numbers to a final MOI of 1 for 24 h. The same settings were established in the absence of bacteria as controls. Additionally, in some wells infected with both strains in the presence or absence of bacteria, a final concentration of 8 ng/ml of fluconazole was added. Fluconazole was dissolved in DMSO, and it was ensured that the final percentage of the organic dissolvent in the wells was below 0.1%.

After 24 h, supernatants and attached cells were collected and vortexed thoroughly. Vaginal cells were treated with 0.5% Triton-X-100 for 5 min to lyse them and release adherent fungal cells. Samples were diluted appropriately with PBS. The diluted samples were plated on YPD plates with 1× PenStrep (Gibco, Thermo Fisher Scientific) and incubated at 37°C for 1 to 2 days until adequate growth for determining the CFU was reached.

### Sequencing.

For the DNA extraction, the strains were grown in YPD cultures for 16 h at 37°C and 180 rpm, and the following protocol was implemented to isolate DNA of high quality. The cultures were centrifuged for 5 min at 4,000 rpm. The pellet was suspended in sorbitol 1 M and centrifuged for 2 min at 13,000 rpm. Then the pellet was resuspended in SCEM buffer (1 M sorbitol, 100 mM Na-citrate pH 5.8, 50 mM EDTA pH 8, 2% β-mercaptoethanol, and 500 units/ml lyticase [MERCK]) and incubated at 37°C for 2 h. Afterward, the samples were centrifuged for 5 min at 13,000 rpm, and the pellet was resuspended in proteinase buffer (10 mM Tris-CL pH 7.5, 50 mM EDTA pH 7.5, 0.5% SDS, and 1 mg/ml proteinase K) and incubated at 60°C for 30 min. Phenol:chloroform:isoamylalkol 25:24:1 was added after the incubation, and the samples were vortexed for 4 min. Then they were centrifuged for 4 min at 13,000 rpm. Then the aqueous phase was transferred to a new tube and a 1:1 volume of cold isopropanol was added. Samples were centrifuged for 15 min at 13,000 rpm. The pellets were washed with 70% ethanol once and centrifuged again for 3 min at 13,000 rpm. After drying, the pellet was resuspended in water and RNase. The genomic DNA was stored at –20°C until sequencing. The sequencing of the clinical strains was done by the company GENEWIZ, using Illumina NovaSeq 2 × 150-bp sequencing and 10 M raw paired-end reads per sample package. Additionally, paired-end reads for non-*petite* clinical isolates were obtained from a previous study (NCBI SRA project SRP099102 [43]). All reads were aligned to the C. glabrata reference genome (version s03-mo1-r06 [12]) using Bowtie2 version 2.4.1. Variants were called from the resulting alignments using the call variants script in bbmap version 38.44 (SOURCEFORGE, 2014) with standard parameters. The resulting variant files were applied to the reference genome by the bcftools consensus function version 1.10.2 (81).

### *In silico* analysis and statistics.

All the results were obtained from at least three biological replicates (indicated in the figure legends). The means and standard deviations of these replicates are shown. Experiments performed with MDMs were isolated from at least three different donors (see figure legends). Data were analyzed using Prism 5 (GraphPad Software, San Diego, CA, USA). The data were generally analyzed using a two-tailed, unpaired Student’s *t* test for intergroup comparisons, if not indicated otherwise.

### Data availability.

The raw sequencing data that support the findings of this study are available in the Sequence Read Archive (SRA) of the NCBI under the accession number PRJNA665484.
