# Supplementary figures and images for: Transient Mitochondria Dysfunction Confers Fungal Cross-Resistance against Phagocytic Killing and Fluconazole
Source: mBio. 2021 Jun 1;12(3):e01128-21. doi: 10.1128/mBio.01128-21 (PMC8262853; doi:10.1128/mBio.01128-21)

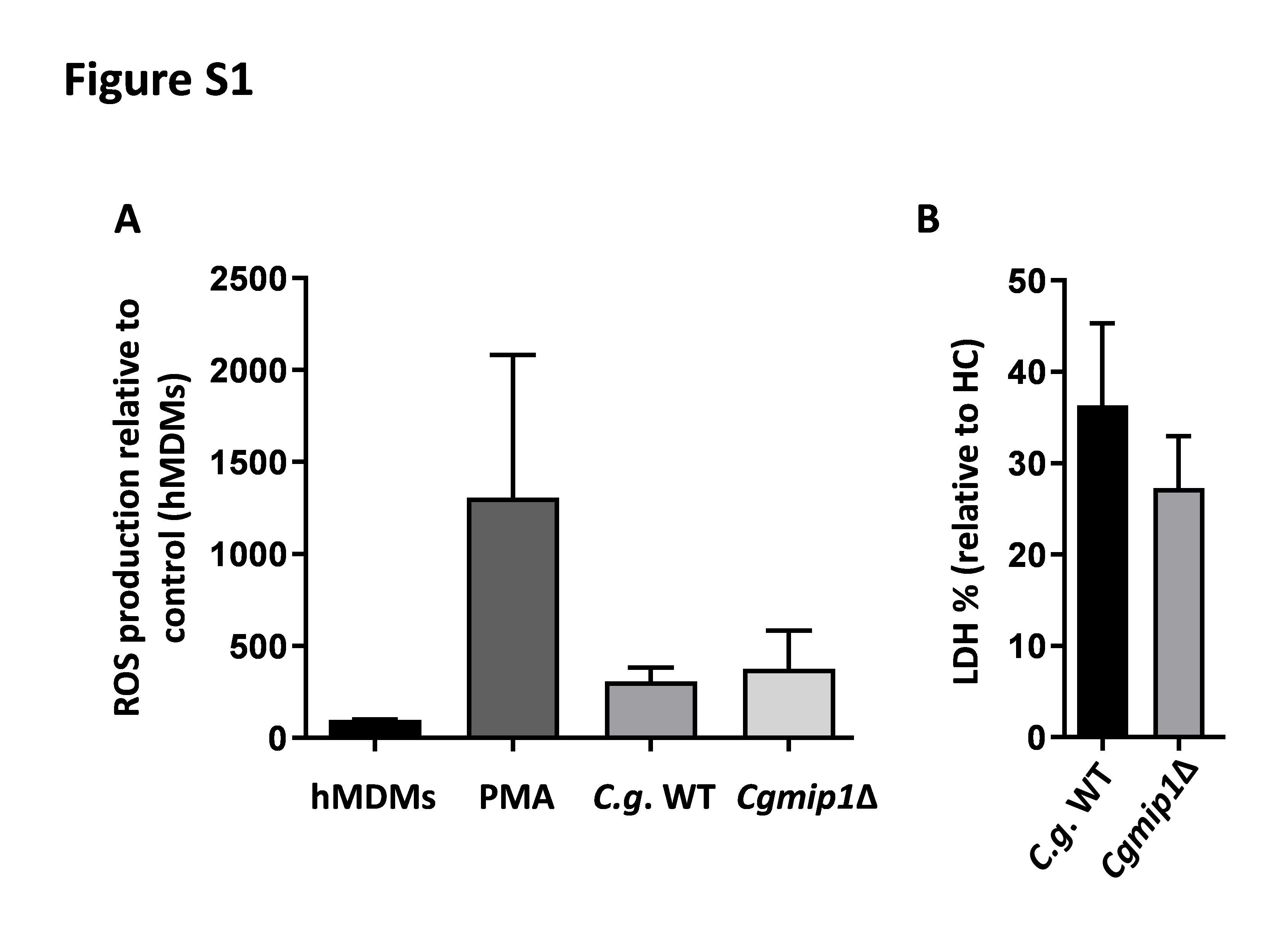

Supplement: FIG S1 [file mbio.01128-21-sf001.tif]

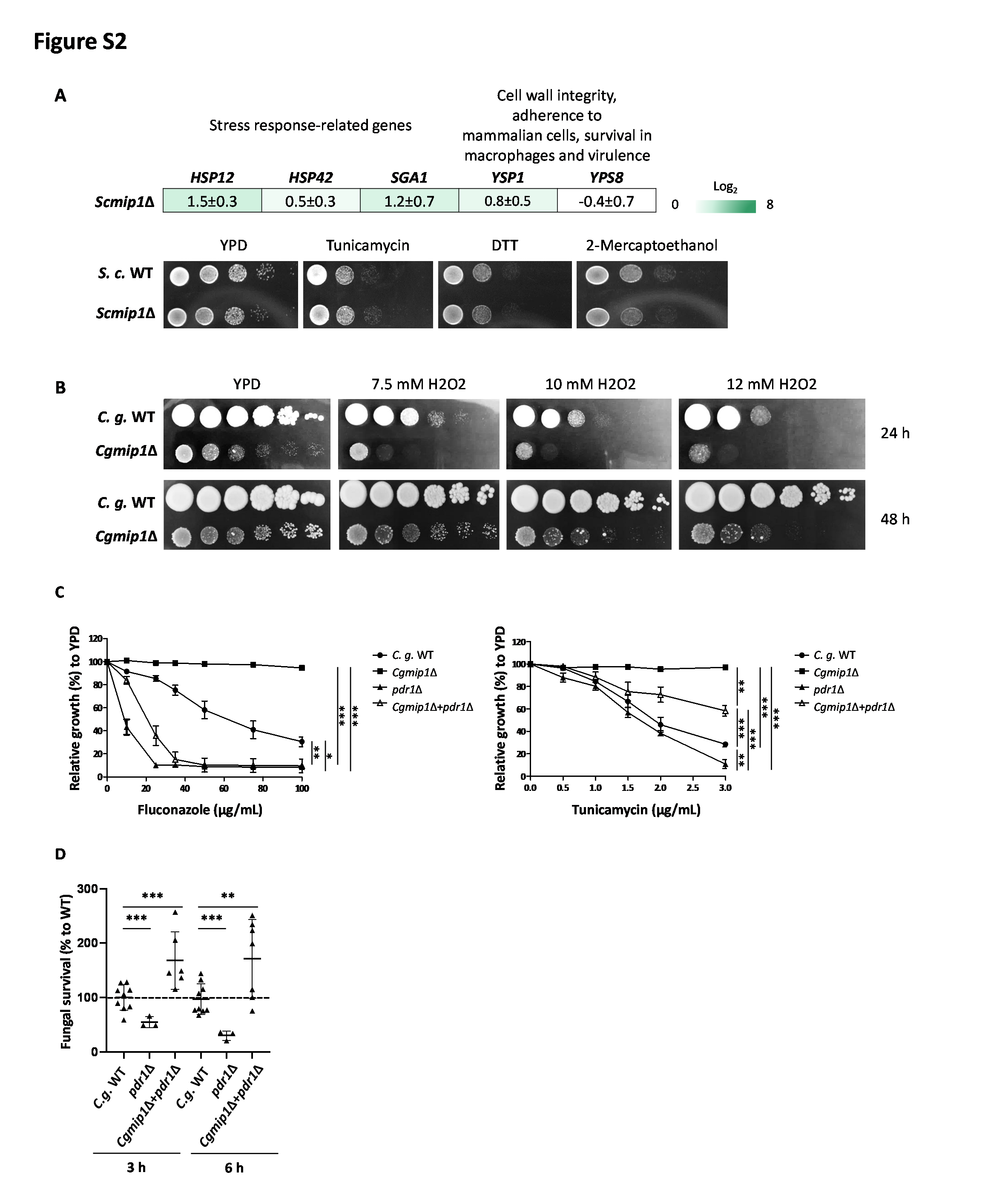

Supplement: FIG S2 [file mbio.01128-21-sf002a.tif]

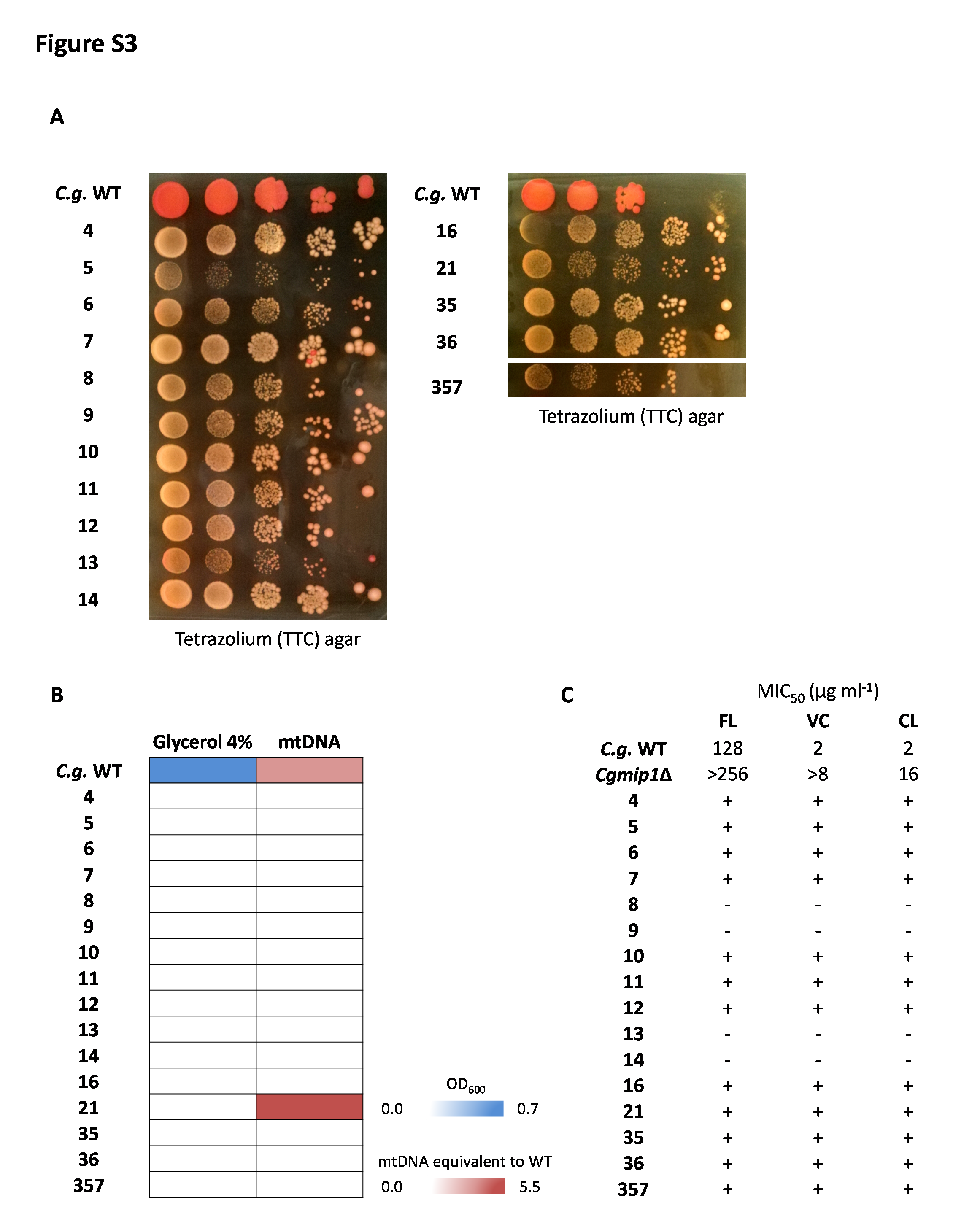

Supplement: Supplementary file 3 [file mbio.01128-21-sf002b.tif]

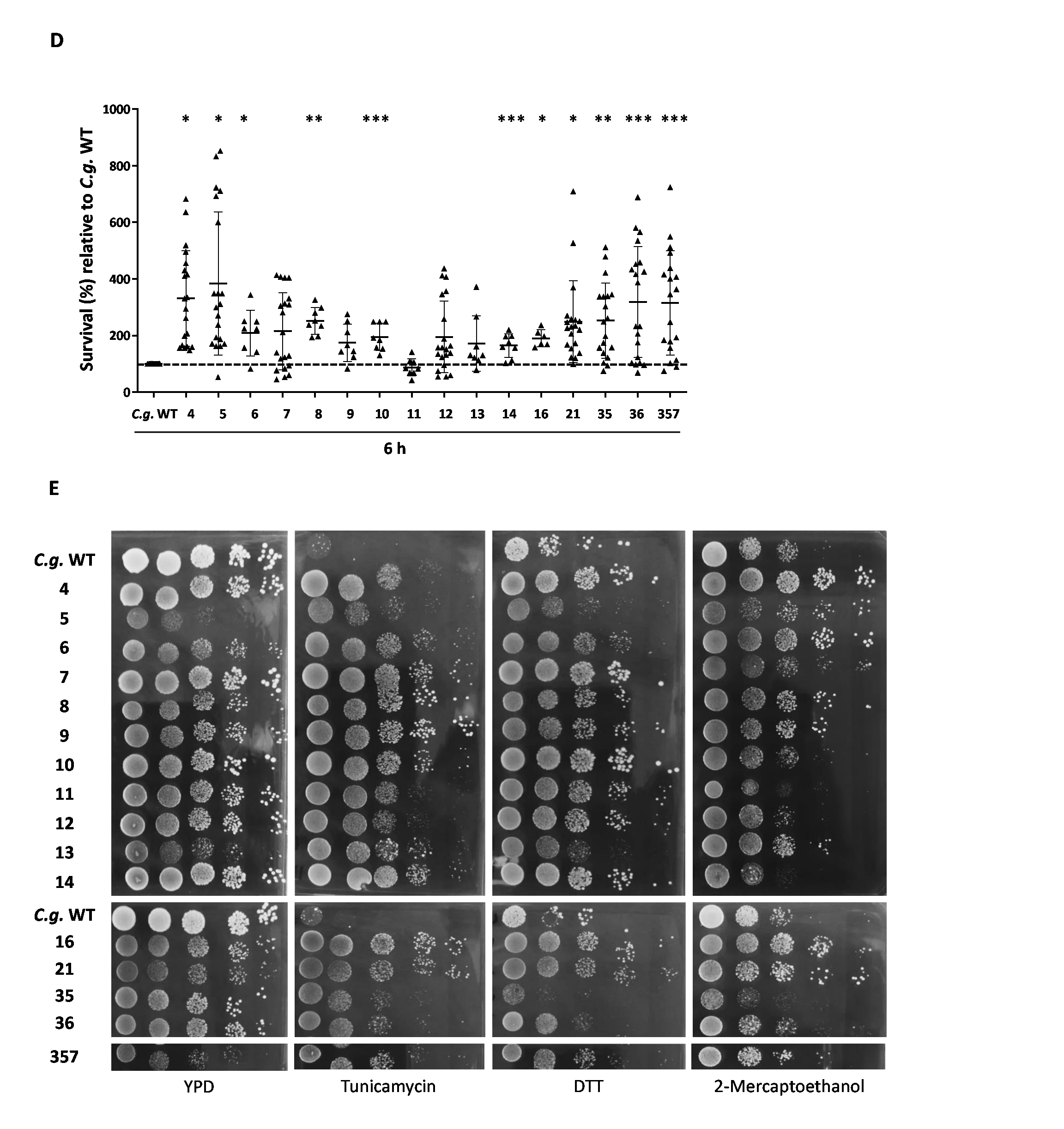

Supplement: Supplementary file 4 [file mbio.01128-21-sf002c.tif]
