# Supplementary material for: Transient Mitochondria Dysfunction Confers Fungal Cross-Resistance against Phagocytic Killing and Fluconazole
Source: mBio. 2021 Jun 1;12(3):e01128-21. doi: 10.1128/mBio.01128-21 (PMC8262853; doi:10.1128/mBio.01128-21)
Supplement: TABLE S1 [file mbio.01128-21-st001.docx]

| **Sample** | **Specimen** | **Sex** | **Age** | **Medical precondition** | **Disease** | **Immunosuppression** | **Antifungal therapy** |
| --- | --- | --- | --- | --- | --- | --- | --- |
| 4 | urine sample | female | 50-55 | right heart failure with pulmonary hypertension | foreign body infection (atrial catheter with KNS) +  pleural empyema (*E. cloacae*) | none | none |
| 5 |  |  |  |  |  |  |  |
| 6 | urine sample | female | 50-55 | Endometriosis | pyelonephritis with urosepsis | none | none |
| 7 | blood culture | female | 35-40 | Crohn's desease | sigmaperforation mit peritonits, *C. glabrata* candidemia | biologicals, cortison high dose | caspofungin |
| 8 |  |  |  |  |  |  |  |
| 9 |  |  |  |  |  |  |  |
| 10 |  |  |  |  |  |  |  |
| 11 | expectorate | male | 30-35 | cystic fibrosis | Exacerbation | none | none |
| 12 |  |  |  |  |  |  |  |
| 13 |  |  |  |  |  |  |  |
| 14 | urine (bladder catheter) | female | 80-85 | cardiac bypass graft | compartment syndrome | none | none |
| 16 | punctate liver abscess | female | 75-80 | pancreatic adenocarcinoma with arterial erusion | abdominal abscess with liver necrosis | chemotherapy | caspofungin |
| 21 | abdominal punctate | male | 80-85 | colon adenocarcinoma, asthma b. | anastomotic leakage with adjunct peritonitis | none | none |
| 35 | sternocleidomastoid abscess membrane | male | 50-55 | ARDS (acute respiratory distress syndrome) due to COVID 19, chronic bronchitis | cervical abscess due to *C. glabrata* and catheter related mycosis | none | caspofungin |
| 36 |  |  |  |  |  |  |  |

**Table S1**
