# Supplementary material for: Transient Mitochondria Dysfunction Confers Fungal Cross-Resistance against Phagocytic Killing and Fluconazole
Source: mBio. 2021 Jun 1;12(3):e01128-21. doi: 10.1128/mBio.01128-21 (PMC8262853; doi:10.1128/mBio.01128-21)
Supplement: TABLE S2 [file mbio.01128-21-st002.docx]

| **Name** | **Genetic background** | **Source** |
| --- | --- | --- |
| *C. glabrata* reference strain | ATCC2001 | Dujon *et al*., 2004 |
| *Cgmip1*∆ | ATCC2001 | This study |
| *Cgmip1*∆+*pdr1*∆ | ATCC2001 | This study |
| *pdr1*∆ | ATCC2001 | This study |
| Macrophages-derived *petites*: MO-1-3 | ATCC2001 | This study |
| Fluconazole-derived *petites*: FL-1-3 | ATCC2001 | This study |
| 4-14, 16, 21, 35, 36 | Clinical isolates | Institute for Hygiene and Microbiology.  Julius- Maximilians- University, Würzburg |
| M17 , EF0313Blo1, EF1521Blo1, EB1114Mou, BG2, EF2229Blo1, EG01004Sto, CST110, M6, P35_3, P35_2, EF1117Blo1, EF1535Blo1, EF1620Sto, EF0616Blo1 , EF1237Blo1 | Clinical isolates | Carreté *et al*., 2018 |
| 357 | Clinical isolates | National Reference Center for Invasive Fungal Infections (NRZMyk) |
| BPY40/41 | Clinical isolates | Ferrari *et al*., 2011 |
| *S. cerevisiae* reference strain | BY4741 | European Saccharomyces Cerevisiae Archive For Functional Analysis (EUROSCARF) |
| *Scmip1*∆ | BY4741 |  |

**Table S2**
